# 'Fight the parasite': raising awareness of cystic echinococcosis in primary school children in endemic countries

**DOI:** 10.1186/s13071-022-05575-2

**Published:** 2022-12-02

**Authors:** Francesca Porcu, Cinzia Cantacessi, Giorgia Dessì, Maria Francesca Sini, Fahad Ahmed, Lia Cavallo, Francesca Nonnis, Katherine Gibson, Cecilia Varcasia, Gaelle Joanny, Antonio Scala, Claudia Tamponi, Antonio Varcasia

**Affiliations:** 1 Laboratory of Parasitology, Veterinary Teaching Hospital, Department of Veterinary Medicine, University of Sassari, Sassari, Italy; 2 Department of Veterinary Medicine, University of Cambridge, Cambridge, UK; 3 Free University of Bozen, Bolzano, Italy

**Keywords:** Cystic echinococcosis, *Echinococcus granulosus*, Health communication, Public engagement, Edutainment

## Abstract

**Background:**

Cystic echinococcosis (CE) is a widespread zoonosis and a significant economic concern and cause of morbidity in humans. A scarcity of education on the sources of CE infection and containment measures is considered to be a key factor responsible for persistent transmission within communities. Recently, edutainment approaches have captured the attention of health education (HE) professionals due to the benefits of integrating cognitive and emotional learning processes.

**Methods:**

A study was carried out in Sardinia, Italy, between 2020 and 2022, amid the SARS-Covid-19 pandemic. The project, designed to involve primary school children (via remote or face-to-face learning depending on the evolving Covid-19 containment measures) consisted of four distinct phases: (i) creation of material for school children and teachers focused on cystic echinococcosis; (ii) pre-intervention evaluation of CE knowledge (i.e. True False Don’t Know [TFD] pre-intervention questionnaire based on CE-related knowledge and practices); (iii) edutainment activity (e.g. interactive lessons enhanced by the comic booklet and the “Fight the parasite” cartoon video, hands-on educational activities and drawing activities on CE); and (iv) post-intervention evaluation of CE knowledge (via TFD post-intervention questionnaire [same questionnaire as used for the pre-intervention assessment] on CE-related knowledge and practices) and on-site edutainment tour in primary schools taking part to the project.

**Results:**

The percentage of correct answers increased from 65% for the questionnaire administered pre-intervention to 87.9% for the same questionnaire administered post-intervention (*χ*^2^ = 648.12, *df* = 1, *P* < 0.0001), while the percentage of uncertain answers (i.e. ‘I don’t know’) decreased from 23% pre-intervention to 5% post-intervention (*χ*^2^ = 603.44, *df* = 1, *P* < 0.0001). These differences indicate a significantly enhanced understanding of CE among participating school children after the intervention.

**Conclusions:**

The results of the present survey indicate that the use of digital educational tools, the use of video animations as a model for science communication, as well as other participatory teaching methods, enabled children to retain key knowledge of the routes of CE transmission and ways to prevent it.

**Graphical abstract:**

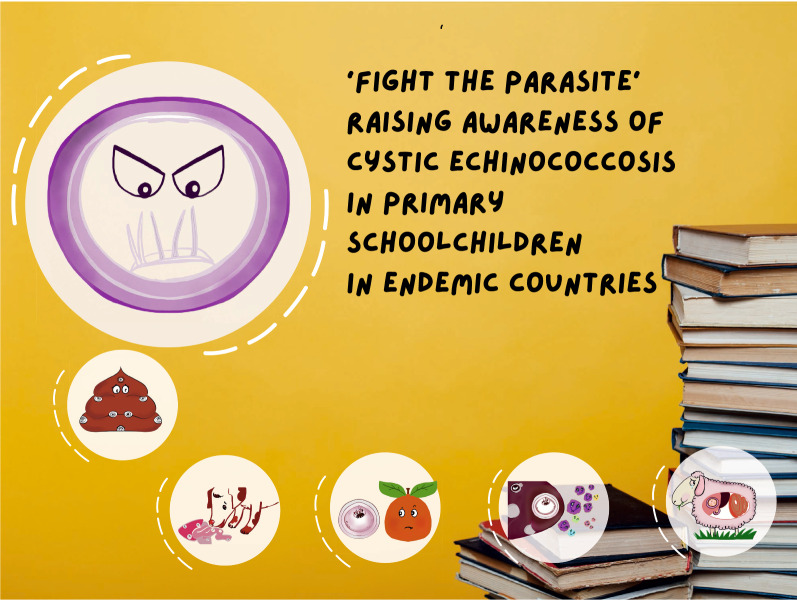

**Supplementary Information:**

The online version contains supplementary material available at 10.1186/s13071-022-05575-2.

## Background

Cystic echinococcosis (CE) is a widespread zoonosis caused by the larval stage of the tapeworm *Echinococcus granulosus *sensu lato (*E. granulosus *s.l.). The disease is listed as one of the most severe parasitic diseases in humans and is prioritized by the WHO as one of the 17 most neglected tropical diseases [1]. The infection causes significant morbidity in humans, with over 1 million cases reported worldwide [2]. According to recent CE burden estimates, about 300,000 disability-adjusted life years (DALYs) and approximately US$200 million are spent annually for the treatment of CE in humans [3]. Dogs and wild canids frequently act as definitive hosts harboring the adult stages of this parasite [4]. Eggs are shed in the feces of the definitive host and thus excreted into the environment. Subsequently, eggs are accidentally ingested by suitable intermediate hosts, which include several species of herbivorous and omnivorous mammals. Humans may also serve as accidental hosts following close contact with infected canids or the consumption of fruits and vegetables contaminated by the parasite eggs [5, 6].

Extensive livestock farming, the presence of numerous stray or shepherd dogs, unsupervised home slaughtering and improper disposal of carcasses are among the most important factors underlying the persistence of CE in endemic areas [7].

However, human behavior (e.g. man-dog relationships and home slaughtering practices) remains a key element determining continued transmission in these areas; consequently, health information and health education (HE) have long been deemed necessary to achieve successful CE control [8, 9] The integration of HE in school-based programs is recommended by the WHO [9]. Indeed, it has been reported that negative health behaviors starting from childhood may persist through adulthood; thus, modifying the behavior of children through HE programs has the potential to significantly lower risky health practices later in life [10]. HE is therefore an effective strategy and a key tool by which to achieve short- to long-term control of CE [11]; in fact, the positive effects of HE have already been validated in principle [12].

Within the broad concept of HE, ‘edutainment’ has garnered the attention of HE professionals over the last several decades. Edutainment is an educational strategy based on ‘warm cognition,’ a branch of study that recognizes the interaction between cognitive and emotional processes in learning [13]. Edutainment integrates entertainment, education and pedagogy [14] and takes advantage of educational videos, cartoons and comic books to communicate complex scientific topics to children [15].

Even though a number of HE programs have been established as part of CE control programs, very few edutainment initiatives have been carried out so far. Thus, in this study, we sought to design and complete an edutainment program for primary school children in an area of Italy (Sardinia) endemic for CE. This health educational project, entitled “Fight the parasite,” seeks to develop effective teaching materials and HE techniques adaptable to CE endemic regions worldwide [14]. The project also aims to determine the level of knowledge of school children on routes of infection and behaviors that may halt transmission of CE prior to and following participation in the edutainment program.

## Methods

The project was carried out from 2020 to 2022, amid the SARS-Covid-19 pandemic. Originally, the project was designed to be delivered to primary school children either via remote or face-to-face learning, depending on national and regional Covid-19 mitigation guidelines at the time of the study. The project involved the following steps: (i) creation of material for school children and teachers, including the multilingual educational comic booklet, the teacher’s guidebook for CE, the educational “Fight the parasite” cartoon video and the pre- and post-intervention questionnaires; (ii) pre-intervention evaluation of CE knowledge (via a True False Don't Know [TFD] pre-intervention questionnaire based on CE-related knowledge and practices); (iii) edutainment activities (e.g. interactive lessons enhanced by the comic booklet and the “Fight the parasite” cartoon video, hands-on educational activities and drawing session on CE); and (iv) post-intervention evaluation of CE knowledge (via TFD post-intervention questionnaire [same questionnaire as used for the pre-intervention assessment] based on CE-related knowledge and practices) and on-site edutainment tour in primary schools taking part to the study. The engaging resources were built on the characteristics of a successful resume described by the CDC, Characteristics of an Effective Health Education Curriculum [16].

### Creation of material for schoolchildren and teachers

#### Multilingual educational comic booklet with activities and games

From July 2020 to September 2020, educational comic booklets were created in Italian, English and Spanish, featuring educational games and activities. First, key risk factors of CE infection and preventive behaviors of school children were evaluated in order to develop a HE package that met the needs of the target population [17]. Subsequently, cultural, behavioral, individual, environmental and educational factors that may influence the efforts to control CE infections were considered to transform the assessed risk factors of CE into effective educational messages, as described in Green’s Precede-Proceed model [18]. The interactive booklet was inspired by The Worm Hunters Project [19]. The booklets were created using the graphic design software Canva (https://www.canva.com/) [20] and downloaded as PDF files for distribution to schools. The booklet has been annexed to the present paper (Additional file 1: Text S1) with the purpose to be replicated in any primary school of the world.

#### Teacher’s guidebook for CE

The CE teacher guide (Additional file 2: Text S2) was developed as a digital information tool for education and training in Italian, English and Spanish. The steps followed in producing the teacher’s guidebook included: (i) assessing the situation (i.e. CE state-of-the-art study and literature search); (ii) defining the learning content of the guidebook; (iii) choosing content and references; (iv) writing text transforming the language of the information found in the literature so as to make it accessible to all levels of society [21]; and (v) designing and drawing illustrations using an Apple Pencil (Apple Inc., Cupertino, CA, USA), an intuitive tool for iPad apps, and IPad 6 (Apple Inc.). The booklet was laid out using the Canva graphic design tool [20], with editing and proofreading [22]. The teacher guide (Additional file 2: Text S2) included information on the epidemiology and life-cycle of the parasite in animals and humans, risk factors for infection and suggestions to halt transmission.

#### Development and production of the educational “Fight the parasite” cartoon video

The “Fight the parasite” cartoon video (Additional file 3: Movie file S1) was developed between December 2021 and August 2022. It is 2 min and 22 s in length and consists of color educational animation featuring an entertaining narrative [23] about CE transmission and prevention [15]. The video was created according to the conscious use of visuals in educational planning [24, 25] and developed to favor meaningful learning [26], engagement, HE awareness and decision-making processes [27]. Prior to video development, educational research was performed on health and hygiene practices and behaviors to prevent infection by *E. granulosus*, and on primary risk factors for parasite transmission, then key messages were selected (i.e. main CE prevention strategies, transmission routes) and the story script was drafted and reviewed. The “Fight the parasite” video was developed to achieve the following objectives (according to HE methodology) [9]: (i) cognition: analysis of problems and solutions to describe and provide knowledge of CE and the parasite life-cycle in animals and humans; (ii) planning: design of appropriate solutions in order to achieve parasite eradication; (iii) operation: action and adoption of appropriate behavior to guide end users in any socioeconomic context toward appropriate lifestyles. Over the course of several brainstorming sessions, the text storyline was drafted as a written document including live dialogue and voiceover. In the video, researchers and school children are depicted as the heroes of a special mission aimed at eradicating *E. granulosus*. The storytelling does not refer to contextual persons and is suitable for the language of children as well as their surrounding context, i.e. the cultural and disciplinary basin of reference. The comic strips are synchronized with the script and according to the content and key messages of the project. The subjects are shown as anthropomorphized animals [28], illustrated using the Apple Pencil (Apple Inc.) and Procreate, a raster graphics editor app for digital painting for iPadOS (Savage Interactive Pty Ltd., North Hobart, TA, Australia). The “Fight the parasite” clips were filmed with the visual-effects and post-production technique of chroma key compositing. Graphic resources, professional voiceover dialog, English subtitles and sound were subsequently added and merged using the professional video editing program Apple Final Cut software V. 10.6.3 (Apple Inc.).

### Pre-intervention evaluation of CE knowledge

From March to June 2022, all participants in this study were interviewed using TFD questionnaires based on CE-related knowledge and practices (Additional file 4: Text S3). The classes of school children involved, after agreement with the respective teachers, did not receive any information on CE before our intervention, to avoid bias. Each participating school child was interviewed in the classroom prior to the beginning of the participatory classroom meeting. Each questionnaire included five questions pertaining to knowledge and practices [29] on CE (see Additional file 4: Text S3).

### Edutainment activity

The participating school children were subsequently engaged in a 1-h-long presentation on CE [30]. The activity was led by the members of the research team and focused on CE infection routes, transmission and prevention. The interactive lessons [29] included classroom discussion and hands-on educational activities [15], including use of a light microscope for observation of alcohol-fixed, mounted and stained *E. granulosus* parasites. The educational comic booklet (Additional file 1: Text S1) and the cartoon video (Additional file 3: Movie file S1) were shown and discussed on the multi-media whiteboard. Interventions were supplemented by a drawing session on CE for positive message reinforcement [31]. Some themes or nuclei (i.e. morphology of the *E. granulosus*, biological cycle in intermediate and definitive hosts, characteristics of eggs and their strategies for dissemination in the environment) were presented by means of brief dramatizations [32] and colorful cardboards highlighting key HE messages.

#### On-site edutainment tour in primary schools of a CE endemic region

The edutainment tour was carried out in Sardinia (Italy), a region considered to be hyperendemic for CE based on the annual incidence rate of hospitalized patients being fourfold higher than the national average (6.9/10^5^ vs 1.6/10^5^ inhabitants) [33]. Sardinia is the second largest island in the Mediterranean Sea and hosts  > 40% of the total sheep population in Italy [34]. Studies on the intermediate hosts have revealed infection rates in sheep ranging from 65.3% to 75% [7, 35], followed by cattle (41.5%), pigs (9.4%) [36] and wild boars (3.7%) [37]. Despite the numerous control campaigns that have been carried out over the past 50 years, CE is still one of the most widespread parasitic diseases in Sardinia [35], with serious social and economic consequences [7]. Between March 2022 and June 2022, the edutainment tour covered 14 municipalities located in seven historical regions of Sardinia (i.e. Romangia, Nurra, Goceano, Barbagia, Mandrolisai, Ogliastra and Campidano), including urban areas, rural areas and peri-urban settings where a previous survey had reported CE infections in animals and humans [7]. In collaboration with local educators, as well as school principals and the teaching staff, 14 primary schools were selected for inclusion in this campaign (Table 1).

**Table 1:** Details and location of the Sardinian primary schools involved in the project

Participating schools	School plexus	Number of school children involved per school plexus	Municipality	Province	Google maps
D.D. Is Mirrionis	C. Collodi SP	151	Cagliari	CA	64H7 + HV
SCI 1 Alghero	Sacro Cuore SP	51	Alghero	SS	H858 + HM
SCI Bono	Bono SP	97	Bono	SS	C26J + JF
SCI Bono	Nule SP	30	Nule	SS	F57R + 22
SCI Bono	Benetutti SP	24	Benetutti	SS	F549 + FX
CI Brigata Sassari	Via Togliatti SP	135	Sassari	SS	PHCM + M4H
SCI Atzara	Sorgono SP	39	Sorgono	NU	24H4 + HJ
SCI Baunei	Urzulei SP	15	Urzulei SP	NU	3GR5 + QV
SCI Bitti	Bitti SP	53	Bitti	NU	F9HP + CQ
SCI Bitti	Orune SP	40	Orune	NU	C949 + 6 J
SCI Bitti	Lula SP	22	Lula	NU	FFCP + F7
SCI 2 Monte Attu	Tortolì SP	125	Tortolì	NU	WMG2 + WR
SCI 2 Monte Attu	Lotzorai SP	69	Lotzorai	NU	XMC7 + 52
SCI 2 Monte Attu	Girasole SP	45	Girasole	NU	XM36 + 5F

*D.D.* Direzione Didattica (Didactic Instruction),* CI *Istituto Comprensivo (Comprehensive Institute),* SCI* State Comprehensive Institute,* SP* school plexis

CA, Carbonia; SS, Sassari; NU, Nuoro

### Post-intervention evaluation of CE knowledge

Assessment of school children’s knowledge post-intervention was carried out by administering a follow-up questionnaire which, as mentioned above, was identical to the pre-intervention questionnaire and delivered immediately after the participatory classroom meeting (Additional file 4: Text S3).

## Results

The project involved 14 primary schools and a total of 896 schoolchildren in 67 classrooms (mean ± standard deviation: 13.6 ± 4.8 students per classroom) (Table 1). The mean age of the school children was 8.7 (± 1.2) years.

**Fig. 1:**
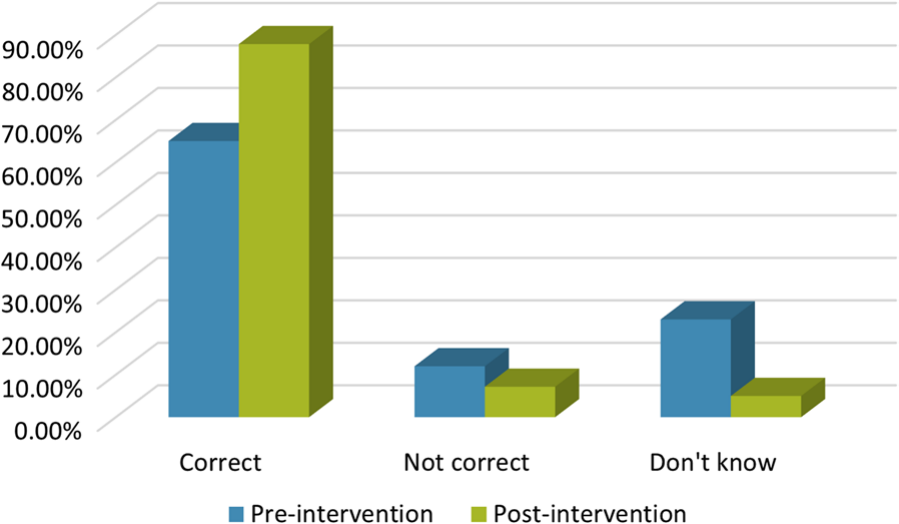
Graphical representation of the improvement in the percentage of correct answers to questions on the questionnaire given pre- and post-intervention. Significant differences were found in the percentage of correct answers, which increased from 65% pre-intervention to 87.9% post-intervention (*χ*^2^ = 648.12, *df* = 1, *P* < 0.0001). Significant differences were also found in the percentage of incorrect (not correct) answers, which decreased from 12% pre-intervention to 7.2% post-intervention (*χ*^2^ = 60.13; *df* = 1, *P* < 0.0001). Also, the percentage of “I don’t know” answers decreased from 23% pre-intervention to 5% post-intervention (*χ*^2^ = 603.44, *df* = 1, *P* < 0.0001)

Comparative analyses of the answers to the questions on the questionnaire when administered pre- and post-intervention revealed that the intervention significantly improved general knowledge of CE, as shown by the percentage of correct answers increasing from 65% (pre-intervention) to 87.9% (post-intervention) (*χ*^2^ = 648.12, *df* = 1, *P* < 0.0001) (Fig. 1). Prior to the intervention, the school children displayed little to no knowledge of the cause of CE (cf. Additional file 4: Text S3). After the intervention, correct answers to the statement “Dogs become infected by *E. granulosus* by eating cooked sheep offals” increased from 42.2% to 75.4% (*χ*^2^ = 204.58, *df* = 1, *P* < 0.0001), from 65.8% to 89.7% for the statement “You could get infected by touching your dog’s feces” (*χ*^2^ = 147.91, *df* = 1, *P* < 0.0001) and from 81.7% to 95.3% for “Washing fruits and vegetables well keeps you protected from the parasite” (*χ*^2^ = 81.63, *df* = 1, *P* < 0.0001). Similarly, correct answers increased from 46.3% to 82.7% for the statement “There is no medicine for dogs against *E. granulosus*” (*χ*^2^ = 259.03, *df* = 1, *P* < 0.0001), and from 88.9 to 96% for “Washing hands after touching a dog can help you not get the parasite” (*χ*^2^ = 31.79; *df* = 1, *P* < 0.0001) (Table 2).

**Table 2:** Comparison of changes in knowledge among school children based on the percentage of correct responses to the questionnaire administered before and after the edutainment activity

Area of knowledge	Responses of school children to the questionnaire					
	Pre-intervention			Post-intervention		
	Correct (%)	Not correct (%)	Don’t know (%)	Correct (%)	Not correct (%)	Don’t know (%)
*General knowledge of parasite biology and transmission routes*						
Dogs become infected by *Echinococcus* by eating cooked sheep offals	42.2	19.0	38.8	75.4	17.9	6.7
You could get infected by touching your dog's feces	65.8	15.3	18.9	89.7	5.8	4.5
*Prevention strategies*						
Washing fruits and vegetables well keeps you protected from the parasite	81.7	7.3	11.0	95.3	2.1	2.6
There is no medicine for dogs against *Echinococcus*	46.3	14.8	38.8	82.7	8.0	9.3
Washing hands after touching a dog can help you not get the parasite	89.0	3.6	7.5	96.0	2.0	2.0

School children clearly associated CE infection with poor eating hygiene, and particularly to inaccurate washing of fruit and vegetables. The percentage of “I don’t know” answers decreased from 23% in the pre-intervention questionnaire to 5% post-intervention (*χ*^2^ = 603.44; *df* = 1, *P* < 0.0001) (Fig. 1).

The educational research and assessment of CE risk factors led to the following key messages being selected [15] for inclusion in the educational tool (see Additional file 1: Text S1; Additional file 2: Text S2; Additional file 3: Movie file S1):

- Wash your hands with soap and warm water after handling dogs and before handling food
- Wash adequately fruit and vegetable with clean water
- Feed your dog well-cooked meat
- Prevent home slaughtering of livestock, especially sheep
- Prevent dogs from feeding on the carcasses of infected sheep
- Treat dogs with effective anthelmintics

These key messages were identified according to the Health Belief Model [15, 38], European Scientific Counsel Companion Animal Parasites (ESCCAP) guidelines [39] and US Centers for Disease Control and Prevention (CDC) prevention measures [40] and incorporated as drawings into key scenes of the educational video cartoon. The video cartoon was completely produced by the research team and can be accessed online (see Additional file 3: Movie file S1).

In several classes we assessed the absorption of these messages through CE-themed drawings post-intervention. In particular, the school children were able to represent the complete life-cycle of the parasite, as well as the main risk factors and behaviors that may lead to infection (see Additional file 5: Movie file S2). Children mainly referred to the images observed under the optical microscope and displayed on the card boards provided during the participatory lesson and reproduced them as faithfully as possible through mnemonic or fantasy images. The teachers’ instruction manual (Additional file 2: text S2; Additional file 6: text S4) provided assistance to the teachers, facilitating their role as educators during the meeting, and represents a resource to further explore the topic with school children during teacher-student contact hours.

## Discussion

The results of the present survey indicate that our strategy, which combines a digital educational toolkit, the use of comic video cartoon as a model for science communication and other participatory teaching methods [9, 15], facilitated children to retain newly acquired knowledge of CE. Comparative analyses of questionnaire results prior to and following the intervention revealed that the participating school children’s knowledge of CE improved by 22.8% and that establishing compliance with all schools was beneficial. The overall percentage of correct answers prior to the intervention (65%) was quite high considering the young age of the children, but it was to be expected given the long-time presence of CE in Sardinia where it has affect the livestock and human populations for decades. Indeed, during the last 60 years there have been several actions aimed at eradicating echinococcosis, including information campaigns which had the support of local broadcasters and, in the late 1980s, a comprehensive “Action Plan for the Eradication of Echinococcosis/Hydatidosis.” For these reasons it is quite possible that school children have heard talk about echinococcosis in their family environment from previous generations.

The significant decrease in uncertain questionnaire answers (“I don't know”) from 23% to 5% is a testimony to the acquisition of self-confidence by the school children toward a new topic, and to the development of critical thinking (yes or no). HE aims to improve health literacy [41], empower end users to access health information and use it effectively and increase autonomy [41]. This, in turn, enhances opportunities for educational attainment and self-reliance, and assists independent citizens to make informed decisions on health and disease and to evaluate the consequences and ethics of their actions on themselves and society [42].

An understanding of fundamental hygiene practices to minimize the risks of CE transmission, such as thoroughly washing fruits and vegetables and washing hands prior to eating and after handling a dog or touching soil, improved substantially post-intervention. This was likely aided by an increased awareness of hygiene procedures implemented during the COVID 19 pandemic. However, the intervention helped reinforce these positive behaviors: these are not issues to be overlooked as habits such as picking vegetables and herbs in the countryside or consuming produce from home gardens without washing them, even if believed to be safe behaviors because the vegetables and herbs have not been treated with pesticides, unfortunately persist.

In line with previous studies, the school children participating in the present study were unaware of the life-cycle of *E. granulosus* [43]. Nevertheless, the children reported knowledge of and/or direct experience with other parasites of companion animals, such as ticks, fleas and roundworms, which serves as a proxy indicator of the importance a given community places on a particular subject [44]. In addition, this emphasizes the importance of conducting surveys during primary prevention projects, as these can help delineate the perception of the school-age population of disease in endemic areas by raising community awareness and/or developing such awareness from childhood [45] which, in turn, encourages active prevention measures [43]. Prior to the intervention, the children were not fully aware of the routes of CE transmission and, in particular, that the consumption of raw offal is a risk factor for dogs (question 1) and thus humans. This lack of knowledge could be justified by the long latency between infection and symptoms in humans (which also appear as nonspecific) [46]. Furthermore, when completing the pre-intervention questionnaire, the majority of children were unaware of the existence of effective drugs against *E. granulosus* in dogs (46.3% of answers correct); in contrast, on the post-intervention questionnaire, the percentage of correct responses significantly increased (82.7%). This difference indicates that the children had become conscious of the fact that there are effective drugs against the tapeworm in dogs and that their administration can prevent CE transmission to sheep and humans.

Addressing the issue of home slaughter without official supervision could be helpful, particularly for children in rural areas. Indeed, these children are usually equipped with a wealth of knowledge of local livestock husbandry, coupled with direct experience of home slaughter and carcass disposal (i.e. abandoned in the countryside, buried, incinerated or fed to dogs either raw or cooked). After children are made aware of proper health behaviors, they can subsequently be entrusted with the role of health messenger [29] for their friends and family members. The acquisition of these skills has high potential [47] for the future of young generations from high agricultural and pastoralist settings, particularly in endemic areas where community-based risk is present [46]. Improved health and educational outcomes in school increases the potential for greater economic benefits for children during adulthood as a result of enhanced career opportunities as well as better physical and emotional health; these effects can be passed down to future generations [48]. Teachers are fundamental to the success of school-based health promotion initiatives, given their roles in educating children on health issues and facilitating the development of health literacy skills through classroom and school activities [49]. Pursuing the goal of improved health literacy will also require more overt alliances between health and education sectors at local, national and international levels emphasizing, for example, the need for improved alliances between WHO and United Nations Educational, Scientific and Cultural Organization (UNESCO), at an international level, and clearer understanding between agencies at the local level [41].

## Conclusions

The authors believe that it is vital to promote broad and multidisciplinary educational approaches to raise awareness of zoonotic diseases, as human beings are built on words, in work and in action-reflection [32]. HE programs in schools should be designed and carried out with input from experts, such as veterinarians, in accordance with the new European animal health law that emphasizes the active role that these professionals should play in raising awareness of the importance of animal and human health [50]. The results of the present study also support the establishment of an echinococcosis HE system, the launching and promotion of school-based HE interventions on CE and the implementation of a gradual and dynamic education model for students [43]. The current framework for the control of CE is mainly focused on prophylaxis, chemotherapy-based control vaccination and HE. The added benefits of including edutainment in programs for the prevention of CE as part of an integrated multicomponent approach for sustainable control must be emphasized. It has been repeatedly shown that additional public health measures, including novel and effective health educational interventions, are needed to establish an atmosphere conducive to the development of good personal hygiene [31]. Edutainment has long been an internationally recognized tool successful in delivering complex ideas and raising awareness of a wide range of topics, particularly among the younger generations, whose actions and choices can greatly reduce the spread of CE. However, short-term interventions are not intended for single use. Healthy behaviors need to be sustained over long time periods to achieve health advantages [18]. Therefore, it would be desirable to implement such programs throughout the school years, in order maximize benefits and monitor long-term results.

## Supplementary Information

**Additional file 1: Text S1.** Educational comic booklet featuring educational games and activities on CE, edited in English and Spanish.

**Additional file 2: Text S2.** Teacher’s guide. A digital information tool for education and training on CE, edited in English and Spanish.

**Additional file 3: Movie file S1. **“Fight the parasite” cartoon video, link and QR code, showing CE transmission and prevention in an entertaining narrative form.

**Additional file 4: Text S3.** Questionnaire to assess students’ knowledge of CE and quiz solutions with the right answers, edited in English and Spanish.

**Additional file 5: Movie file S2. **Link to slideshow of artwork made by children.

**Additional file 6: Text S4. **Instructions for teachers to carry out the project in the classroom.

## Data Availability

The datasets used and/or analysed during the current study are available from the corresponding author on reasonable request.

## Abbreviations

CDC: U.S. Centers for Disease Control and Prevention
CE: Cystic echinococcosis
DALYs: Disability-adjusted life years
ESCCAP: European Scientific Counsel Companion Animal Parasites
HE: Health education
TFD: True False Don’t Know
UNESCO: United Nations Educational, Scientific and Cultural Organization
