# Supplementary material for: 'Fight the parasite': raising awareness of cystic echinococcosis in primary school children in endemic countries
Source: Parasit Vectors. 2022 Dec 2;15:449. doi: 10.1186/s13071-022-05575-2 (PMC9717558; doi:10.1186/s13071-022-05575-2)
Supplement: Supplementary file 1 — Additional file 1: Text S1. Educational comic booklet featuring educational games and activities on CE, edited in English and Spanish. [file 13071_2022_5575_MOESM1_ESM.pdf]

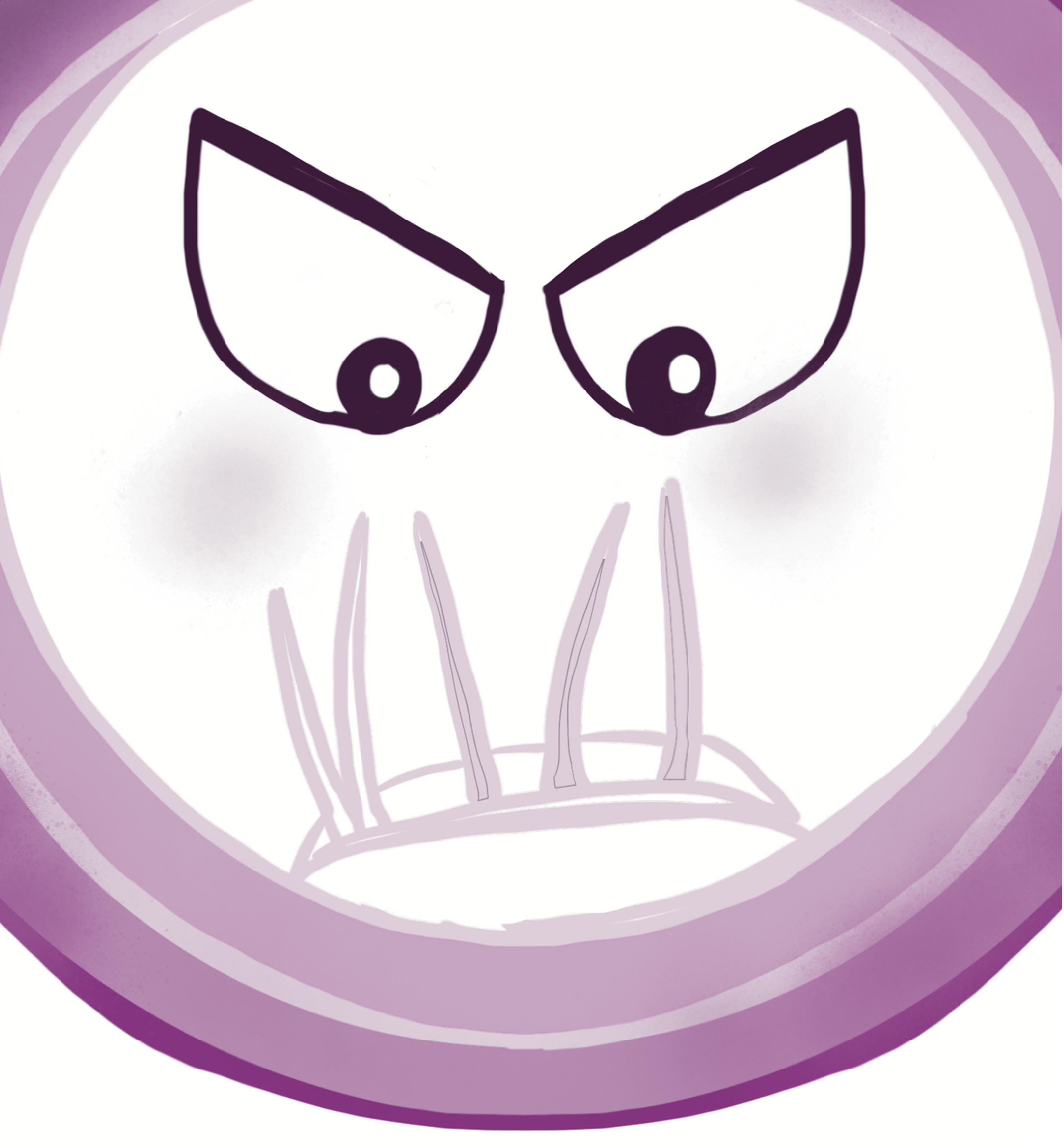

Fight the parasite!

An edutainment project for Cystic Echinococcosis awareness in primary schools of endemic countries

Let the adventure begin!

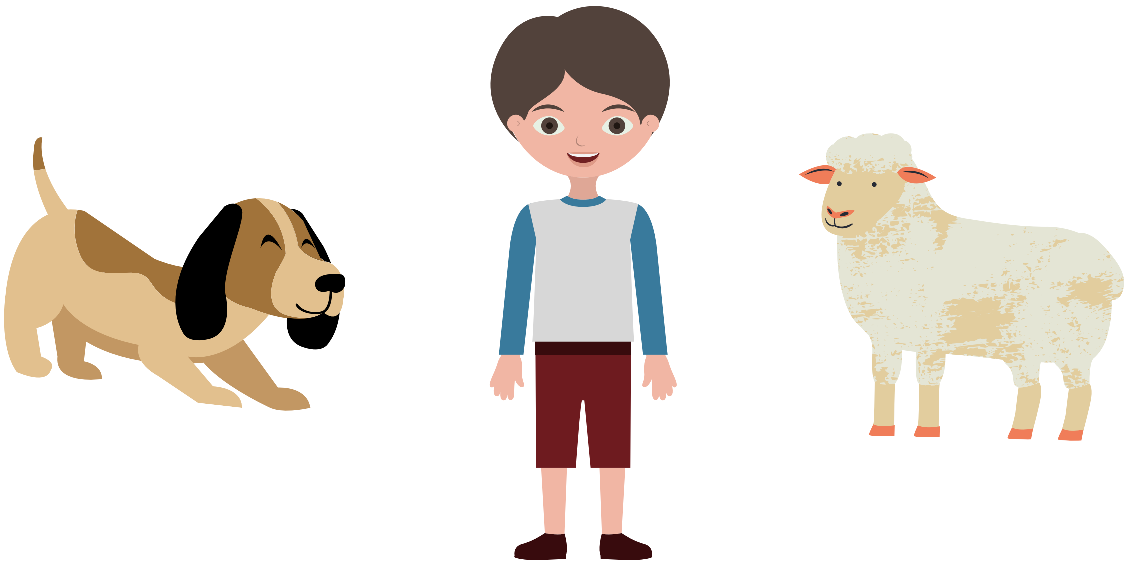

This health education workbook has been developed by Cambridge University Veterinary School, United Kingdom, and Veterinary Parasitology of the University of Sassari, Italy.

This book belongs to:

School:

Your Mission

Hello!
My name is Katherine. I am a veterinary student and I have been learning about a nasty bug which hurts animals and people. Your mission is to help my friend, Francesco, work out how to protect everyone from this horrible bug.

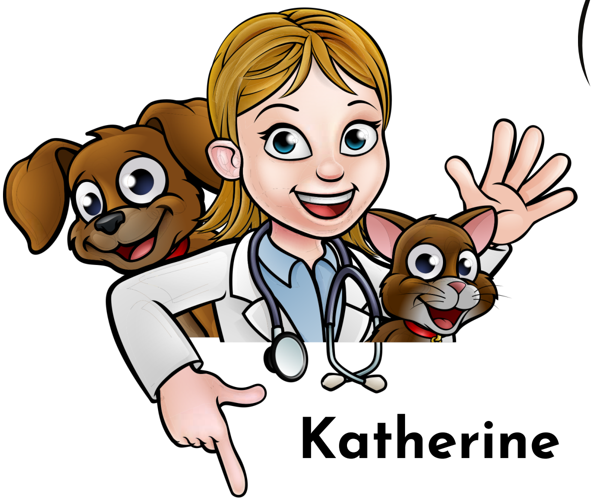

Katherine

I'm Francesco.
Me and my dog, Tricky, really need your help. First we must find out what this nasty bug is. Then we can work out how to stop it.

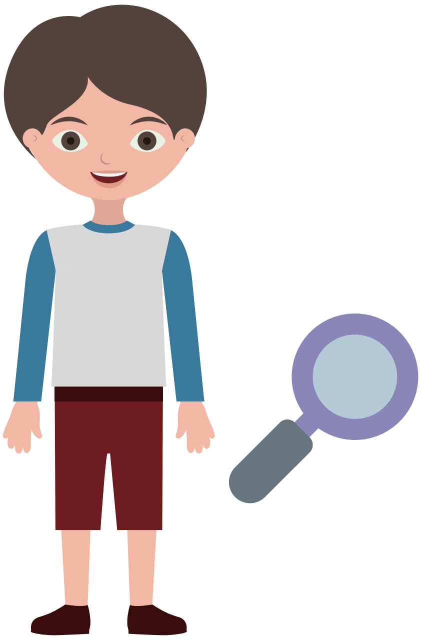

Francesco

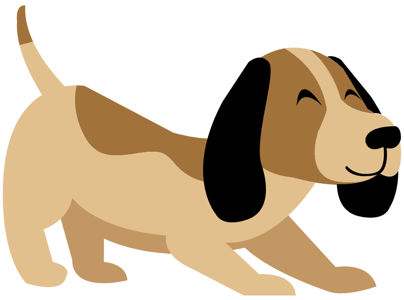

Tricky

Woof! Woof!

Find the nasty bug

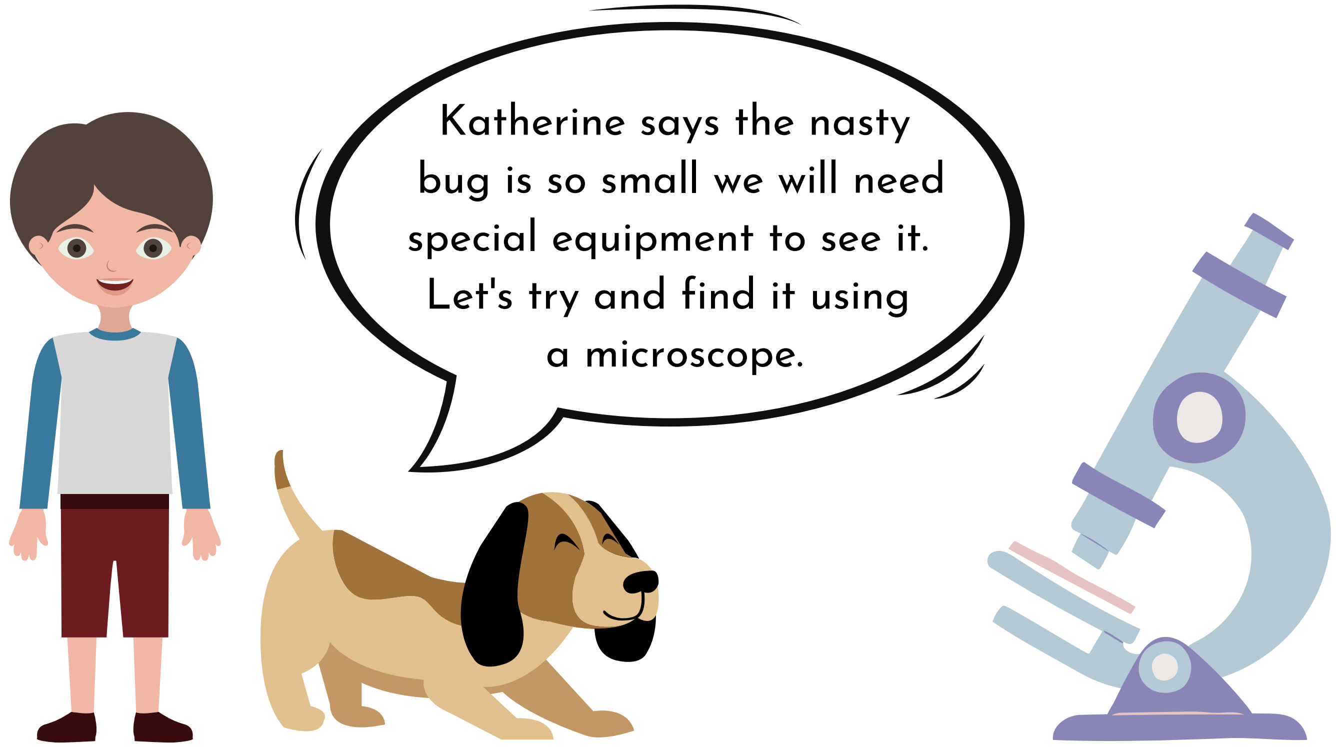

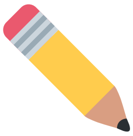

Join up the dots to see what the nasty bug looks like.

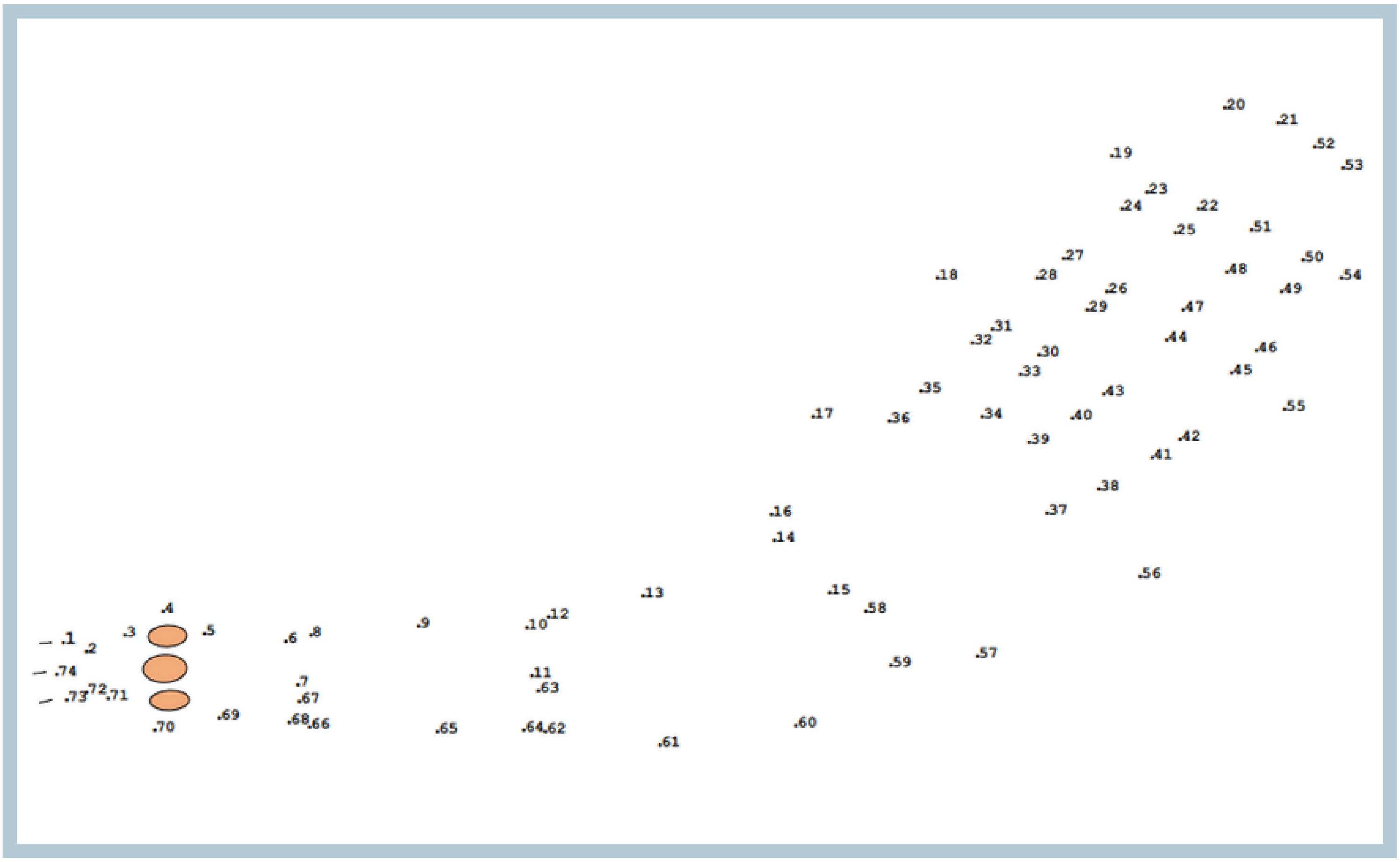

What is the nasty bug?

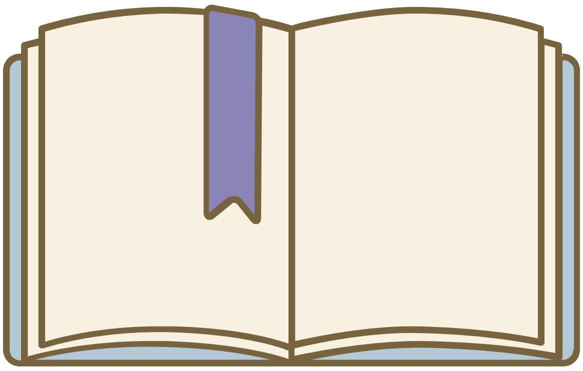

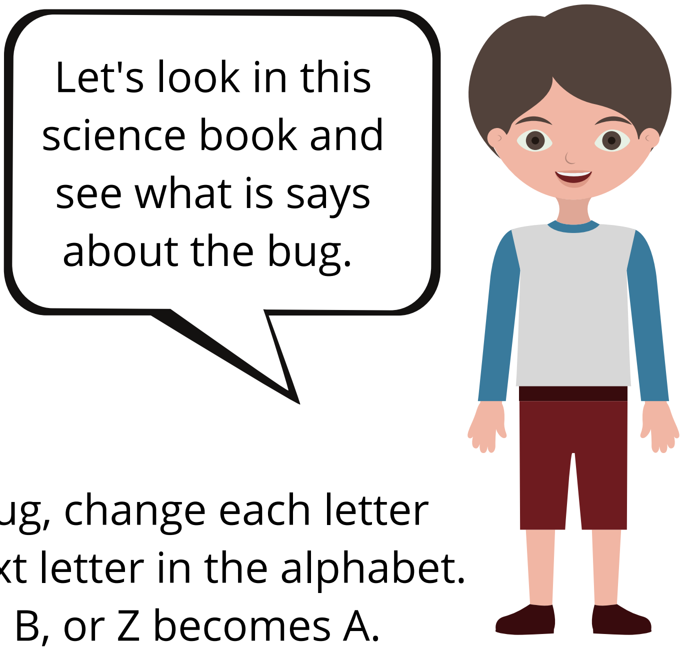

To find the name of the bug, change each letter in the message to be the next letter in the alphabet. For example: A becomes B, or Z becomes A.

S Z O D V N Q L

_ _ _ _   _ _ _ _

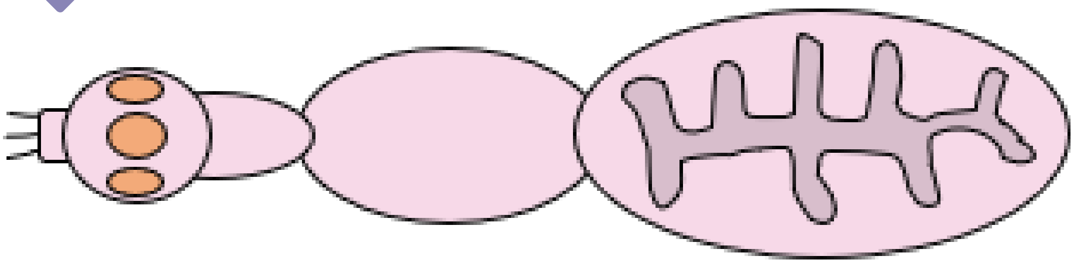

Length: adults are 3-6mm long.

Their eggs are tiny but can survive in difficult conditions.

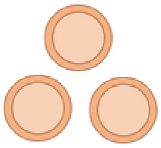

The tapeworm's life

Trace the dotted arrows to follow the tapeworm's life.

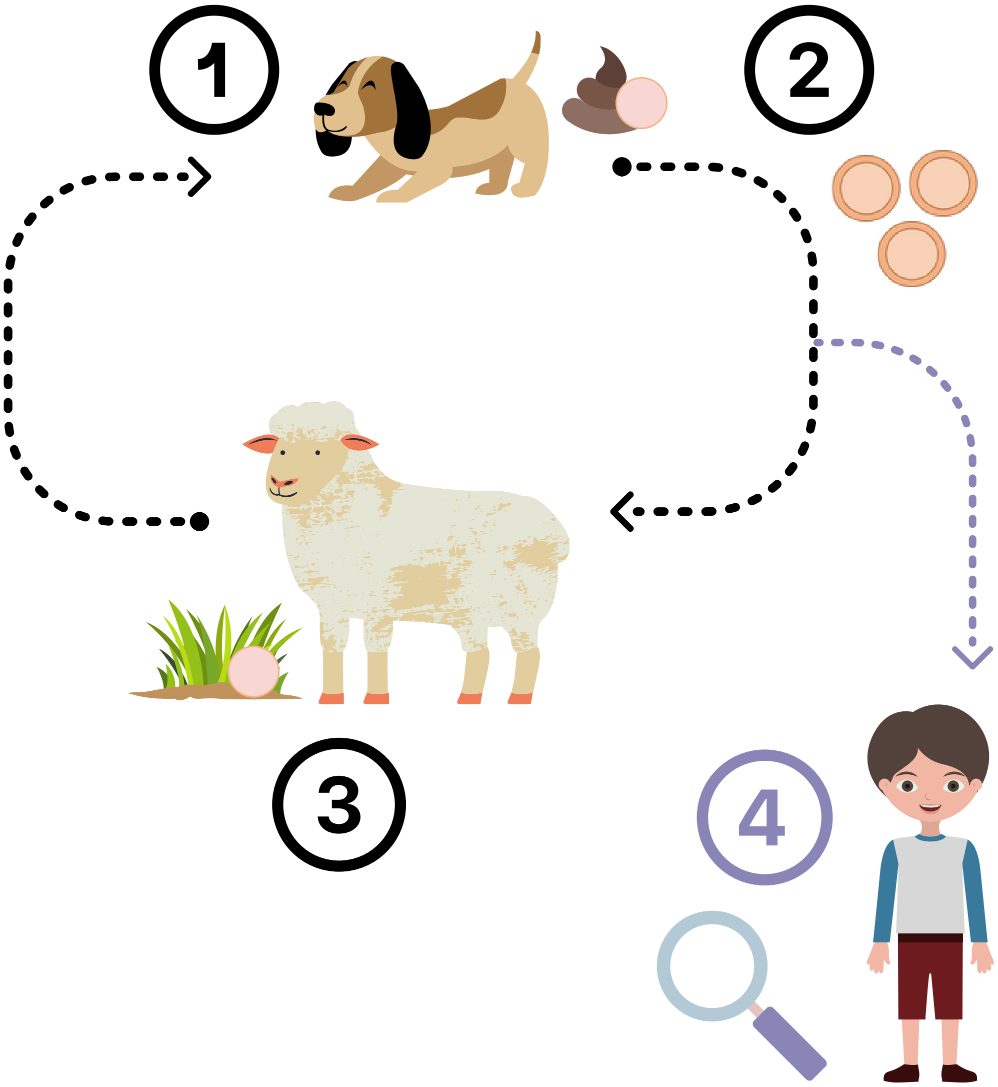

1. A dog eats offals from an infected sheep.
2. The tapeworm lays eggs in the dog's tummy which come out in the dog's poo.
3. A sheep eats grass with eggs which hatch.
4. Humans are accidentally part of the life of the tapeworm when we eat the eggs.

What do tapeworms do?

Tapeworm causes a disease called cystic echinococcosis.
It can make different parts of the body not work well.

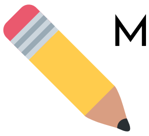

Match the picture of each organ that can be affected to the correct name.

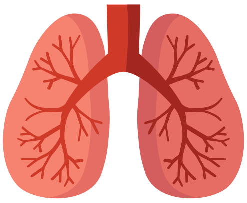	1	Kidney
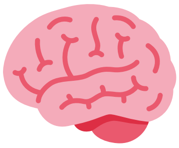	2	Bone
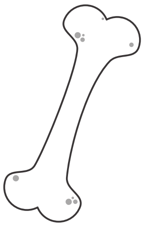	3	Liver
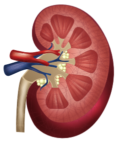	4	Lungs
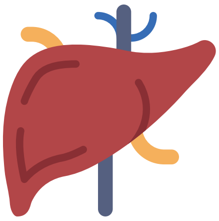	5	Brain

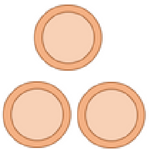

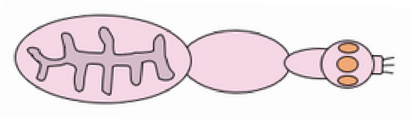

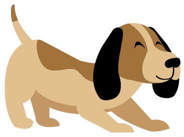

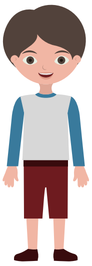

Where in the world?

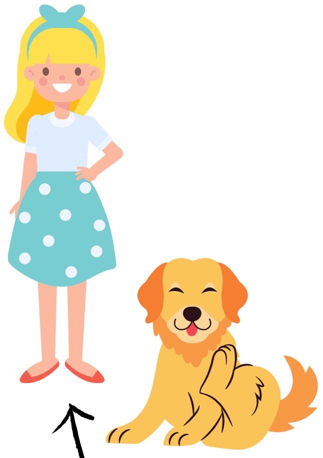

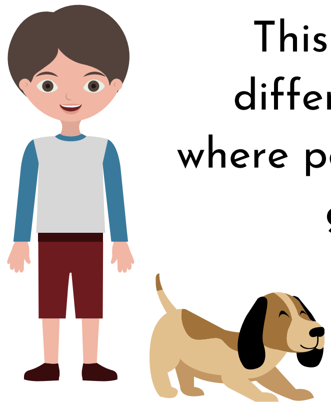

This map shows all the different places (purple) where people and animals can get tapeworm.

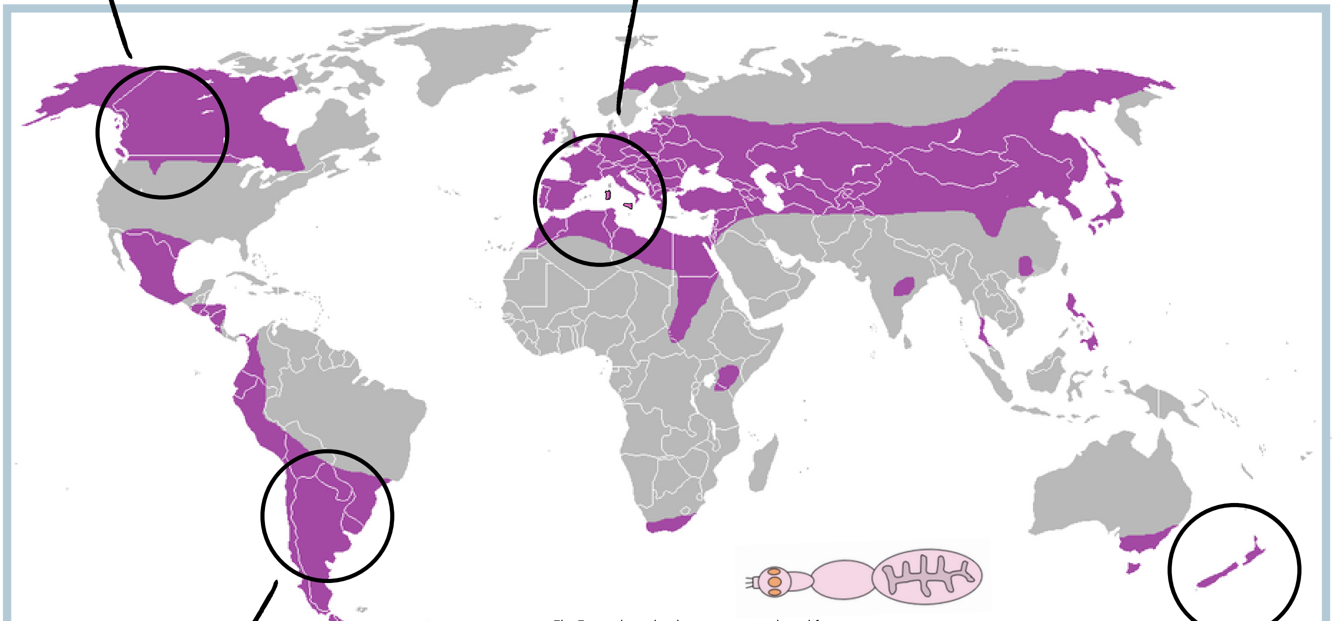

The E granulosus distribution map was adapted from https://commons.wikimedia.org/wiki/File:Echinococcus_granulosus_distribution_map.png.

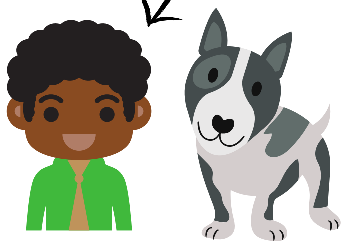

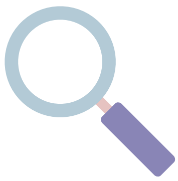

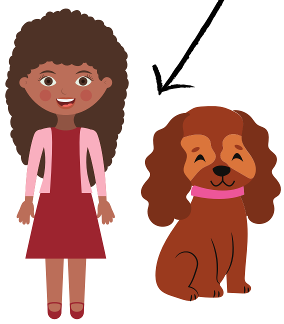

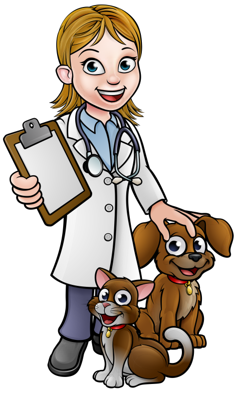

You're doing a great job helping Francesco and Tricky find out about the nasty tapeworm.
Now it is time to work out how we can stop it!

How do we get tapeworms?

Some 'risky behaviours' make it more likely we will get tapeworms.

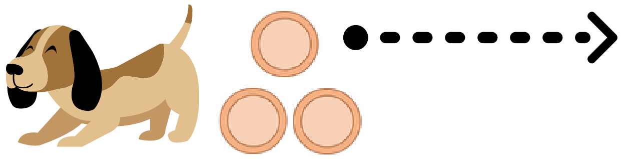

Eggs from dog poo can go into soil, water or food. We can get infected when we...

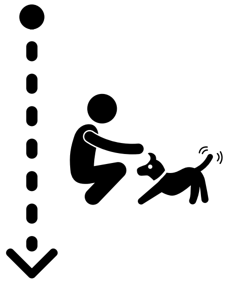

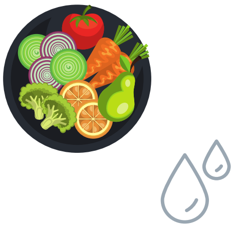

...eat unwashed vegetables.

We can be directly infected when we play with a dog and do not wash our hands.

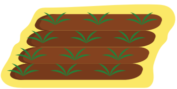

...touch soil and do not wash our hands before we eat.

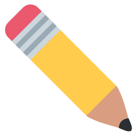

Circle the objects that have tapeworm eggs on them.
How many eggs are there?

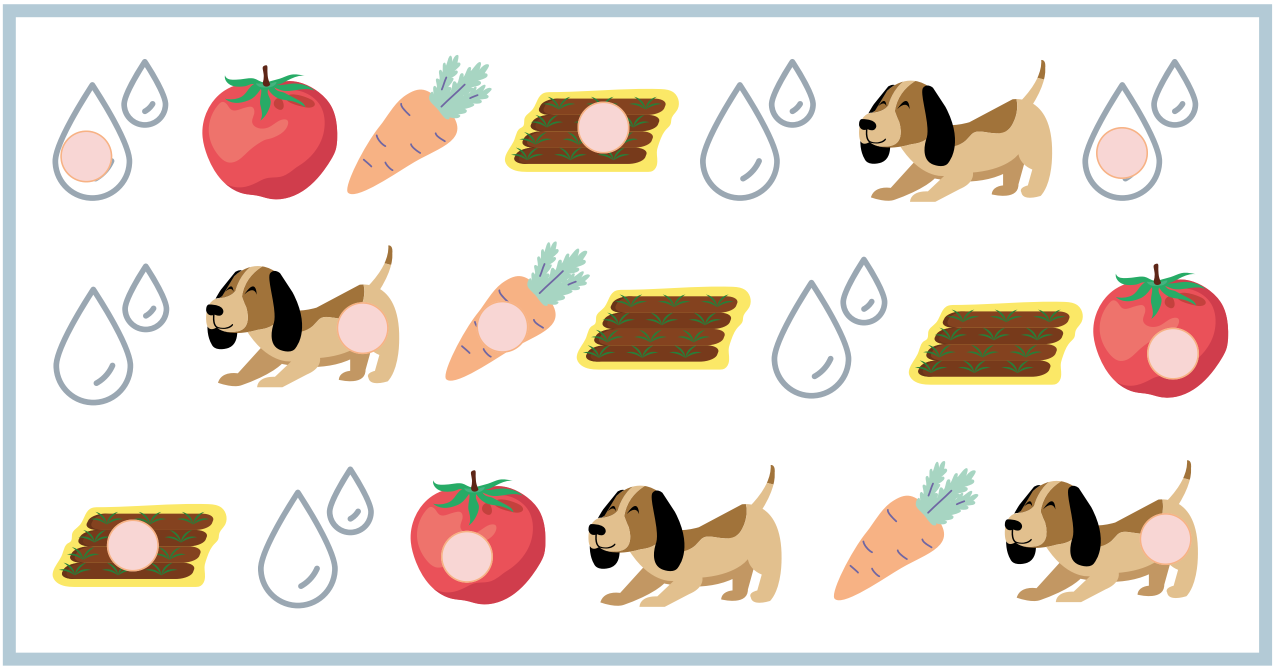

How can we stop tapeworm?

There are certain 'safe behaviours' we can do to protect ourselves from tapeworms. Help Tricky solve the maze to find out what these behaviours are.

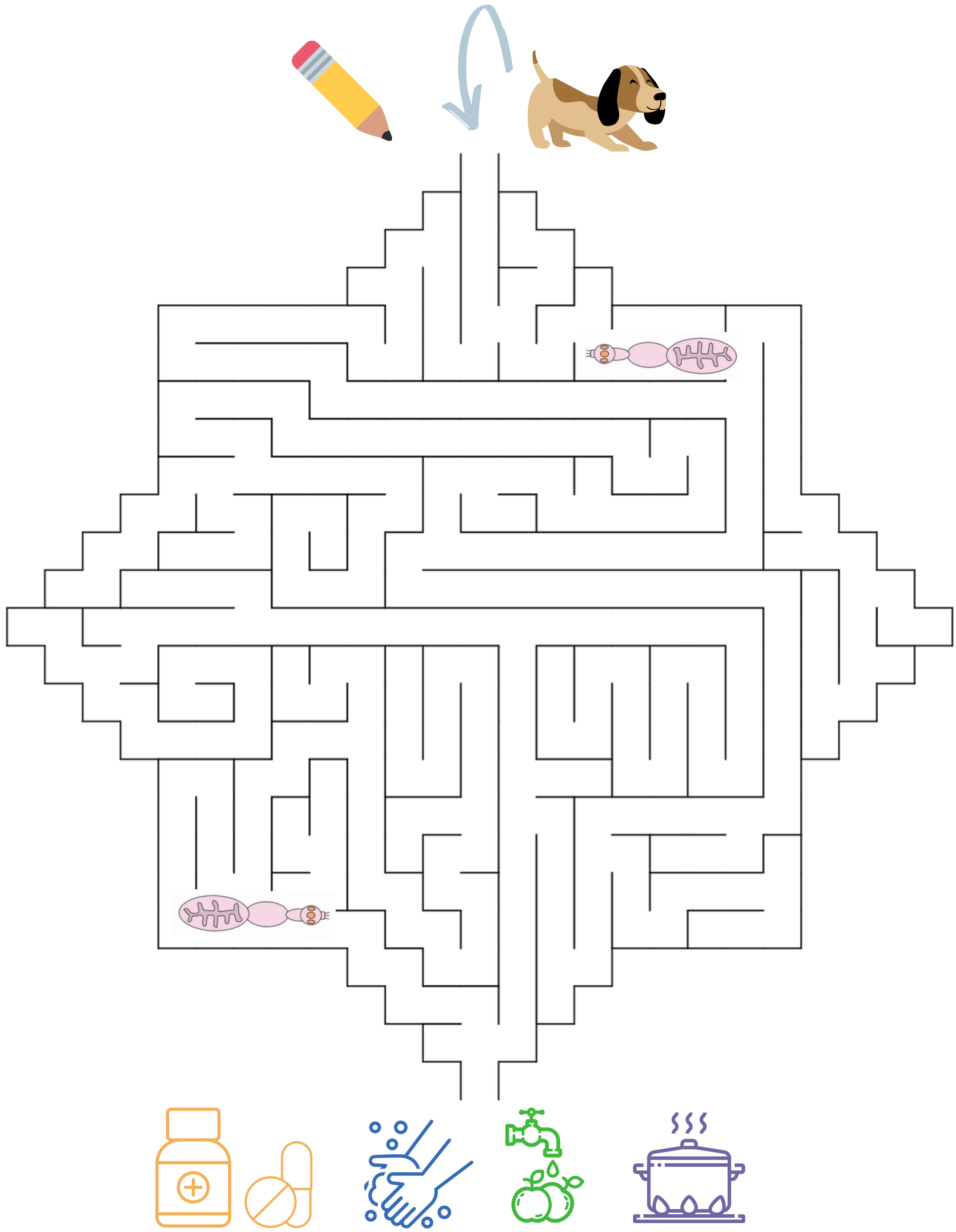

How to stop tapeworm!

How can we stop ourselves getting tapeworm?

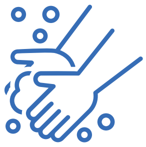

Wash hands carefully after playing with dogs or touching soil where dogs may have pooed

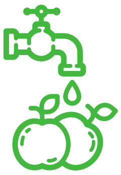

Wash well vegetables and other food with water before eating

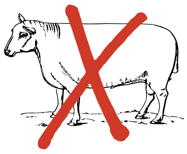

Dogs should not be allowed to eat sheep offal from unsafe slaughter

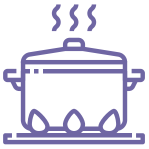

If offal is being fed to dogs it must be boiled for 30 minutes first

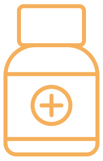

Dogs that have tapeworms should be given medicine

What next?

Can you match the pictures on the left with the ways we can stop ourselves and our dogs getting tapeworm?

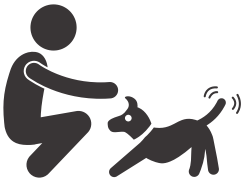

1

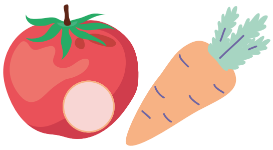

2

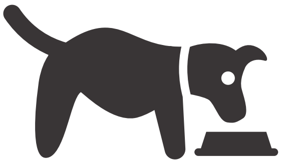

3

4

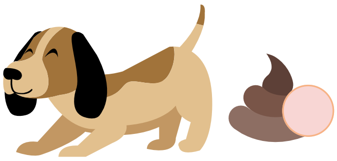

A

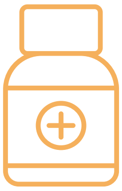

B

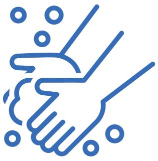

C

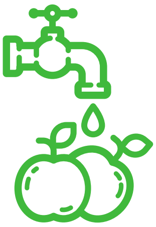

D

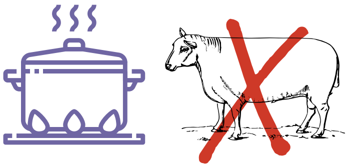

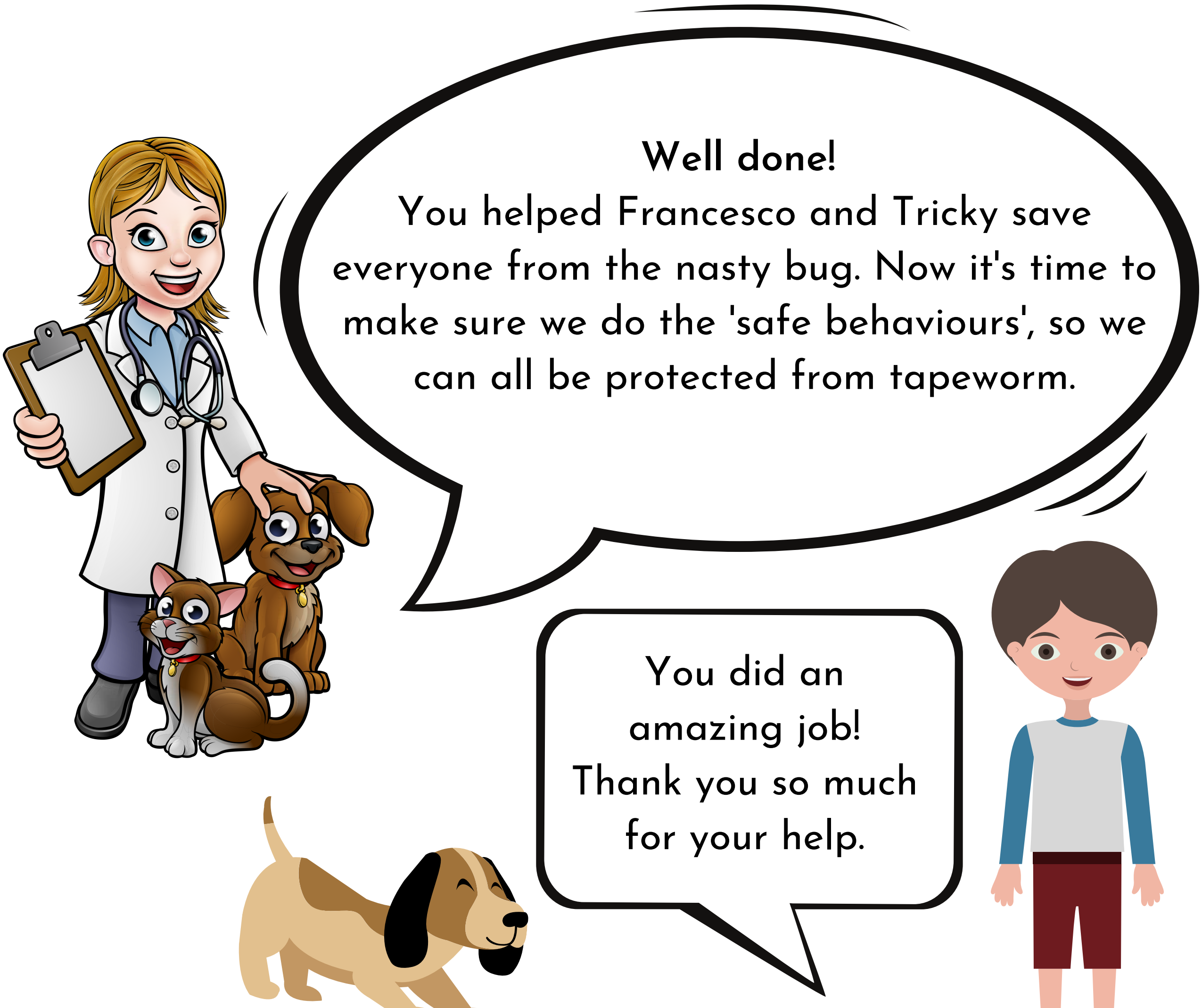

Answers

Page 5: TAPEWORM

Page 7: 1 - Lungs, 2 - Brain, 3 - Bone, 4 - Kidney, 5 - Liver

Page 9: 9 objects have tapeworm eggs

Page 10:

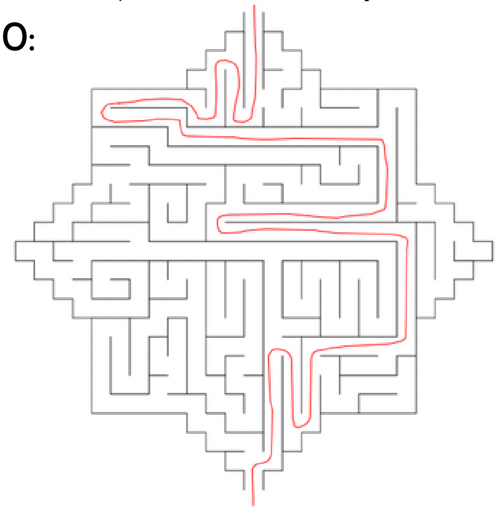

Page 12: 1 - B, 2 - C, 3 - D, 4 - A

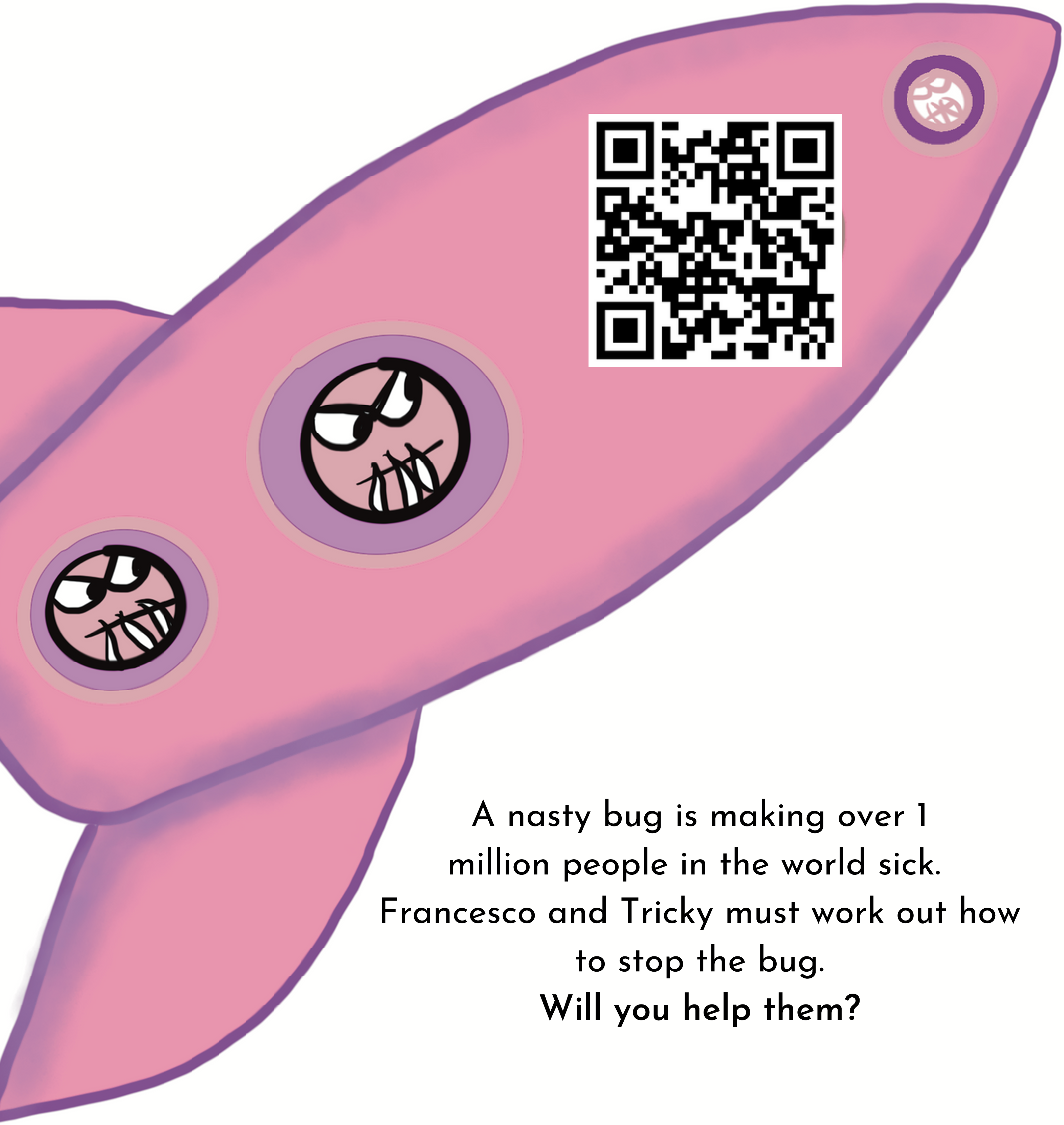

A nasty bug is making over 1 million people in the world sick.
Francesco and Tricky must work out how to stop the bug.
Will you help them?

This project was partially funded by

vetoquinol
ACHIEVE MORE TOGETHER
