# Supplementary material for: 'Fight the parasite': raising awareness of cystic echinococcosis in primary school children in endemic countries
Source: Parasit Vectors. 2022 Dec 2;15:449. doi: 10.1186/s13071-022-05575-2 (PMC9717558; doi:10.1186/s13071-022-05575-2)
Supplement: Supplementary file 2 — Additional file 2: Text S2. Teacher’s guide. A digital information tool for education and training on CE, edited in English and Spanish. [file 13071_2022_5575_MOESM2_ESM.pdf]

Fight the parasite!

An edutainment project for Cystic Echinococcosis awareness in primary schools of endemic countries

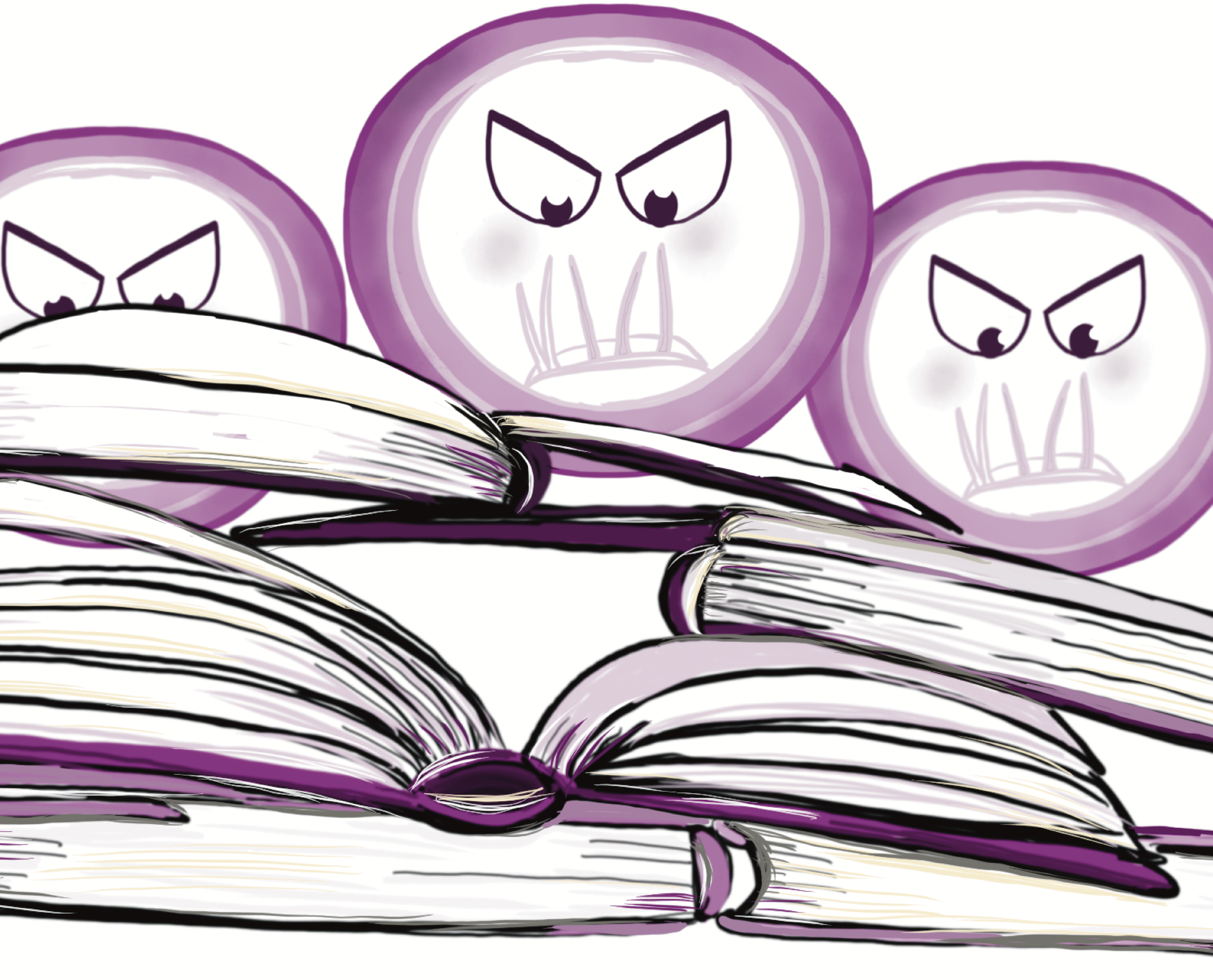

Teacher's guidebook

Dear teacher,
this safety guide has been developed by the Institute of Parasitology and Parasitic Diseases, Department of Veterinary Medicine, University of Sassari and the University of Cambridge, UK.

We carry out research on animal and human parasites in order to adopt the best practices to protect the health and wellbeing of animal and humans under the umbrella of One Health. This is in accordance with objective 3 of the United Nations' 2030 Agenda, which is "to ensure health and well-being for all and for all ages".

Adequate understanding is the foundation of effective prevention, and schools have a unique role in this regard.

A very special thanksgiving

The research team

Laboratories of Parasitology and Parasitic Diseases
of the Department of Veterinary Medicine, Sassari (Italy)
and of the Department of Veterinary Medicine,
Cambridge Veterinary School (UK)

What is cystic echinococcosis?

Cystic echinoccococosis (CE) is a parasitic disease that poses global health concern and is widespread in various regions of Italy, especially in Sardinia island. CE is more common in Mediterranean basin where extensive or semi-extensive farming of livestock (mostly sheep) is practiced. The World Health Organization (WHO) includes human CE in the list of twenty neglected tropical diseases (NTDs) and the list of neglected zoonotic diseases (NZDs), for which an effective control strategy is warranted. The disease effects more than one million people worldwide.
The disease is caused by the larval stage of a very small tapeworm called *Echinococcus granulosus*, which is 2 to 7 mm in length and has an indirect biological cycle, requiring two hosts, one definitive and one intermediate.
The dog represents the most common definitive host of this parasitosis, since it harbors in its small intestine the adult parasites that live by feeding undisturbed on what the dog feeds on. The intermediate host, however, are sheep and humans, in which it causes major health problems. In Sardinia, the average annual hospitalization rate reported is 6.5 per 100,000 inhabitants, with higher prevalence reported in the Province of Nuoro, where the average annual hospitalization rate is 12.2 patients per 100,000 inhabitants (Brundu et al., 2014).

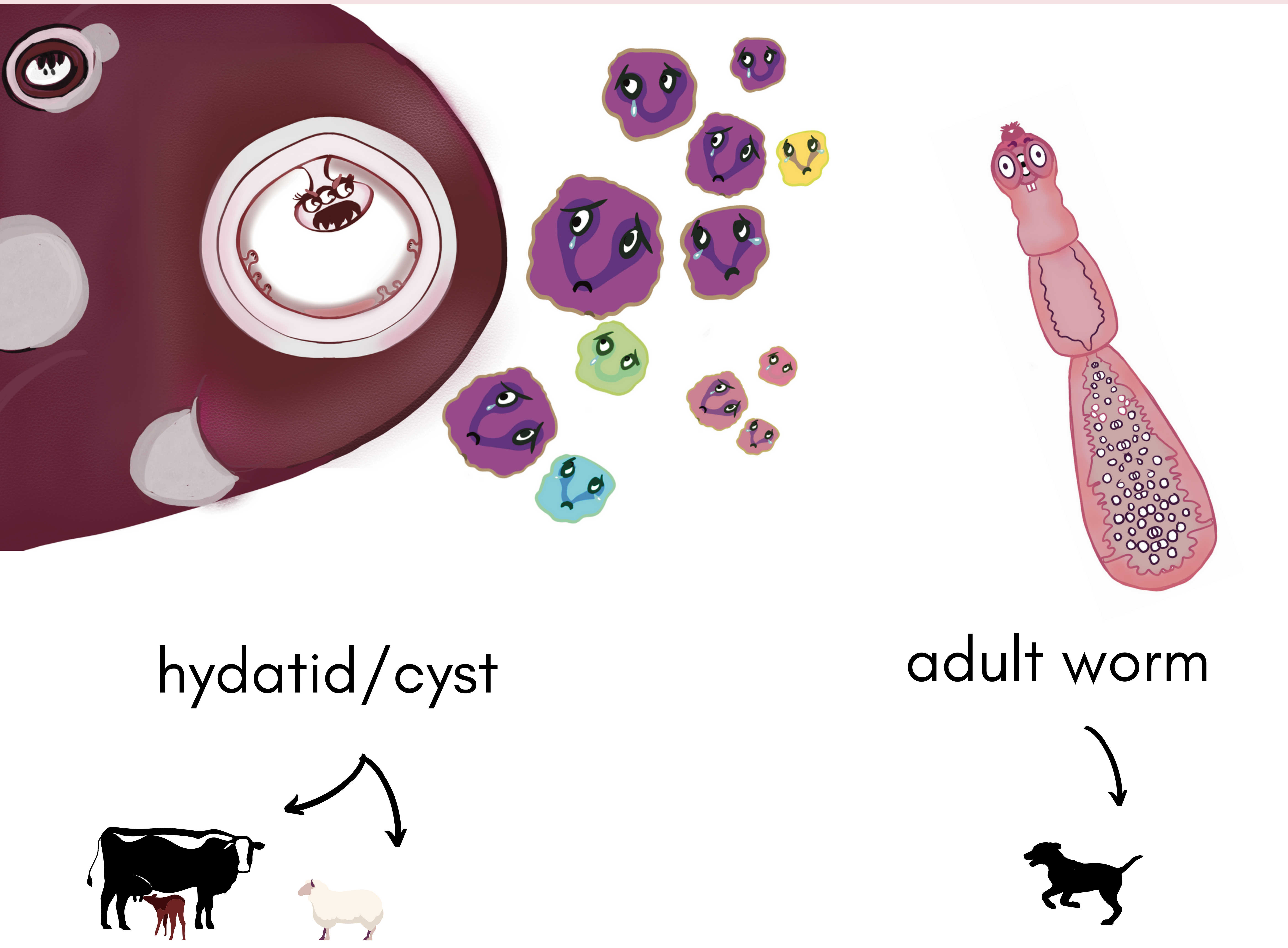

Echinococcus granulosus lifecycle

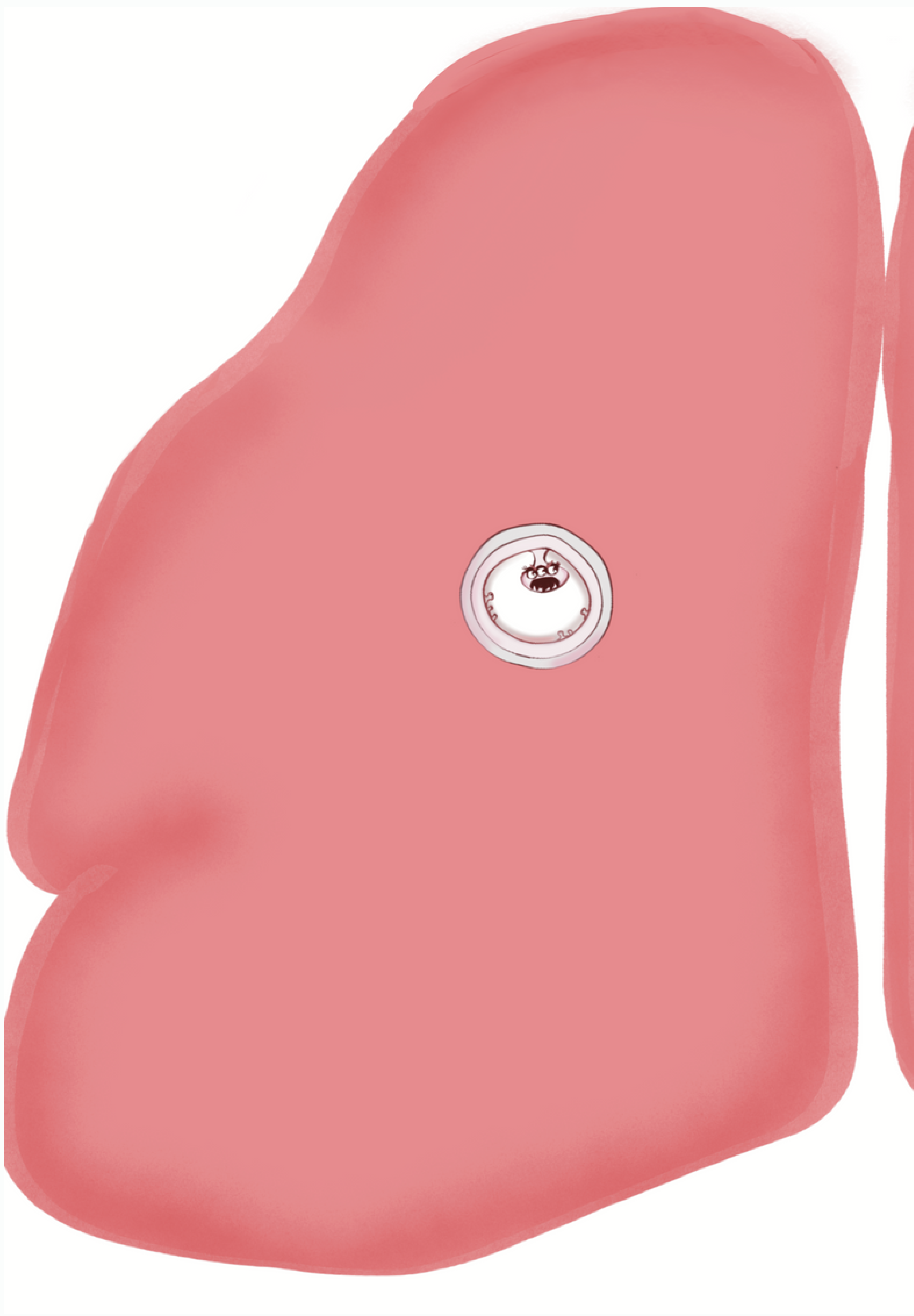

In intermediate hosts, such as sheep, goats, cattles, horses, pigs, and humans, the adult worm is not present, but its larval form develop within certain organs, particularly liver and lungs.
It may take several years for a cyst to reach a size that causes symptoms and pain. The body's immune defenses are ineffective against cysts because of their thick wallr, composed of three concentrically arranged layers: the adventitious membrane, belonging to the host, formed by inflammatory fibrotic tissue; the laminated membrane, intermediate; and a thin membrane belonging to the parasite, called the proligera, which contains ten to thirty infecting forms of the *E. granulosus*, as small as the head of a pin, the protoscolex. The rupture of the cysts can result in fever, allergic reaction, and severe anaphylactic reactions.

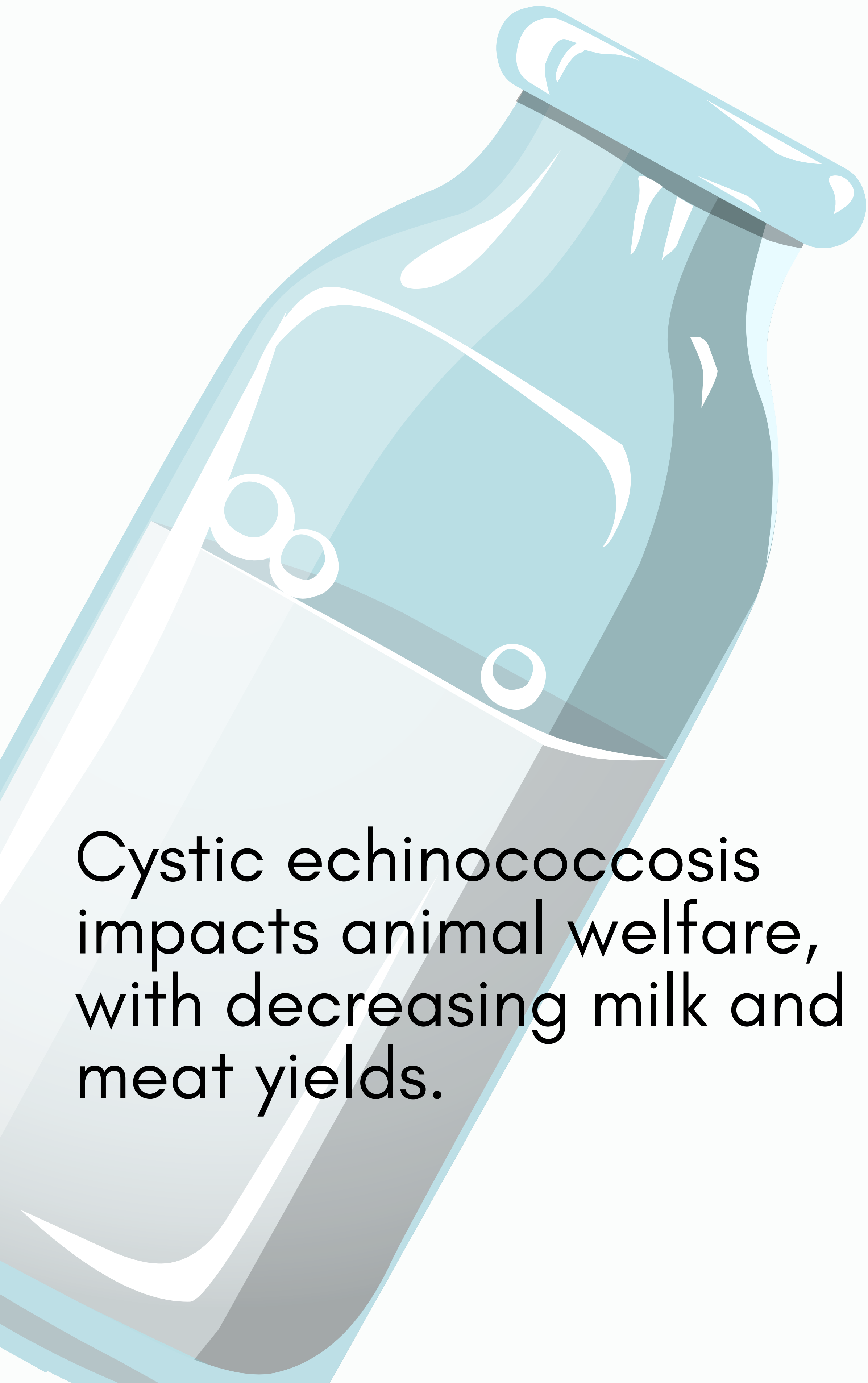

Cystic echinococcosis impacts animal welfare, with decreasing milk and meat yields.

Diagnosis in humans is not easy. It is based on imaging techniques such as ultrasonography, computed tomography (CT), magnetic resonance imaging (MRI), analysis of cystic fluid, or serologic and blood count testing. Symptoms may include fatigue, abdominal tenderness, jaundice and heart failure. Cyst treatment may be pharmacological or surgical, depending on the doctor's diagnosis and the location and size of the cysts.
There are approximately three million sheep in Sardinia, accounting for two-thirds of the Italian sheep population, with the prevalence of cystic echinococcosis between 65.3% and 75.0%. Risk factors for the spread of cystic echinococcosis in Sardinia include the high number of dogs living in close proximity to sheep, stray dogs, socioeconomic factors, cultural conditions and the lack of knowledge of the zoonotic importance of cystic echinococcosis (Scala et al., 2006; Varcasia et al., 2011).

Echinococcus eggs are dispersed into the environment with the feces of dogs, allowing sheep or humans to get infected by consuming poorly washed vegetables and grazing on pastures. Eggs upon entering small intestine convert into oncosphere, a small hooked sphere representing the immature form of the parasite and is encased in an embryonic envelope.

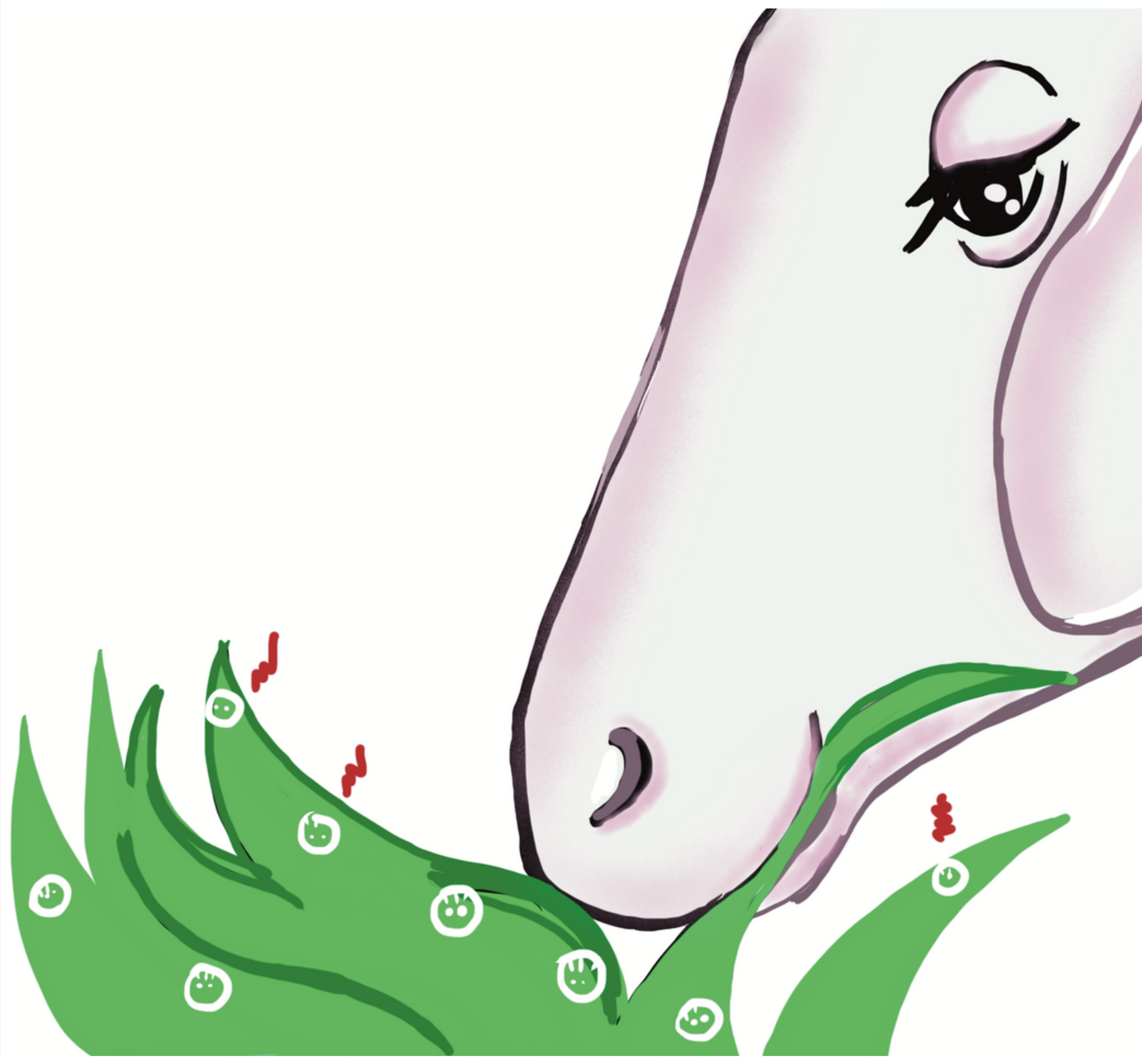

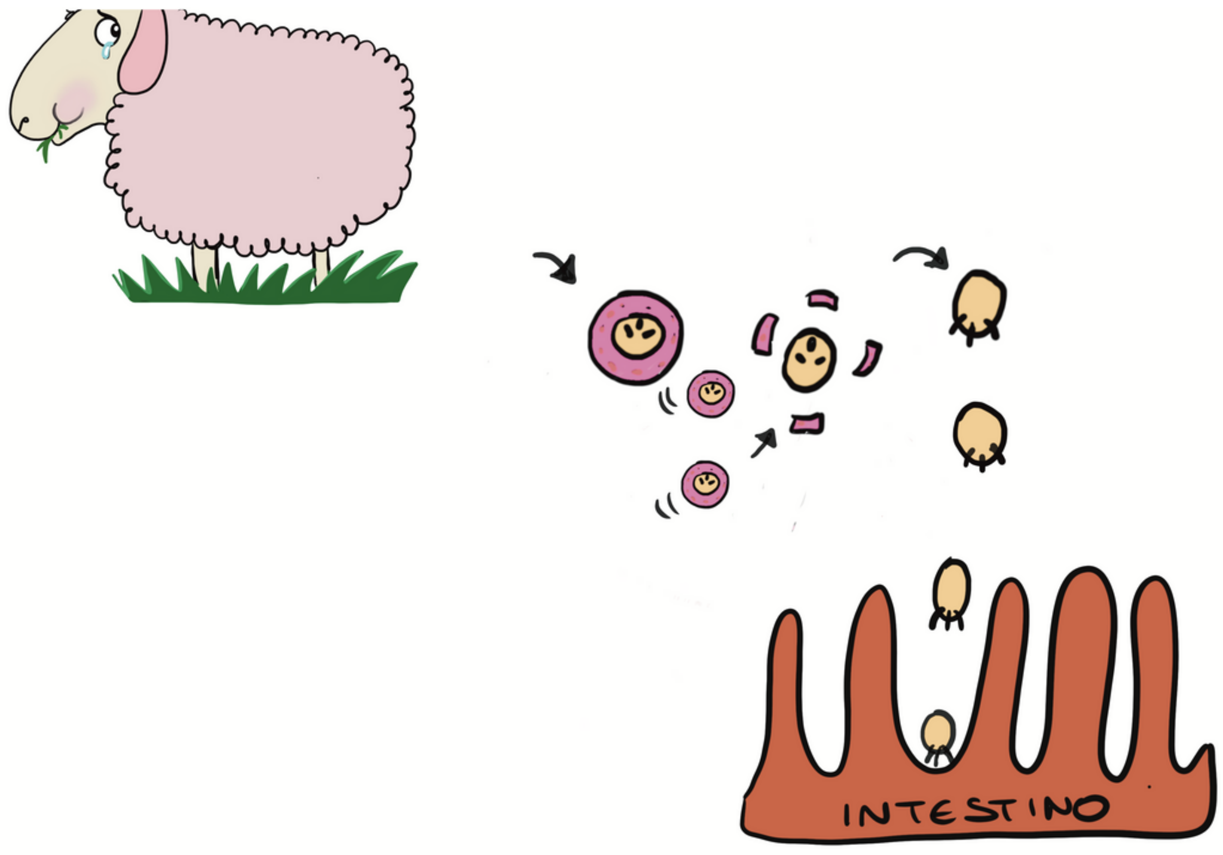

During digestion, pepsin in the stomach and pancreatin in the intestine causes the hatching of the oncosphere and their hooks are able to anchor themselves in the walls of the intestine. After 30-120 minutes, the oncosphere, aided by its hooks, makes its way through the animal's body by lymphohematogenous route to its target organs: liver, lungs, kidneys, spleen, muscles and central nervous system. In these organs, the transformation of the oncosphere into a cyst or hydatid begins. Adult worms are not found in the gastrointestinal tracts of humans.

In tissues, *Echinococcus* oncosphere develop slowly over many years into large fluid-filled lesions called as hydatid cysts. When fully developed they can range in size from an orange to 15-20 cm in diameter. The cysts appear as vesicles with lactescent-white wall and filled with a clear, colorless cystic fluid. Daughter cysts containing numerous small infectious protocysts may form within these cysts. Large cysts may contain over one liter of highly antigenic hydatid fluid and millions of protoscoleces. Sometimes, secondary daughter cysts can arise within or outside the main cysts.

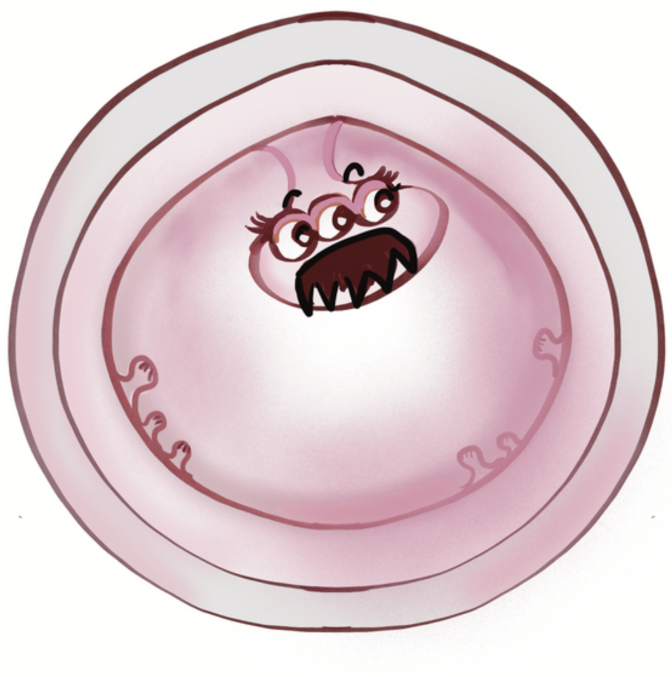

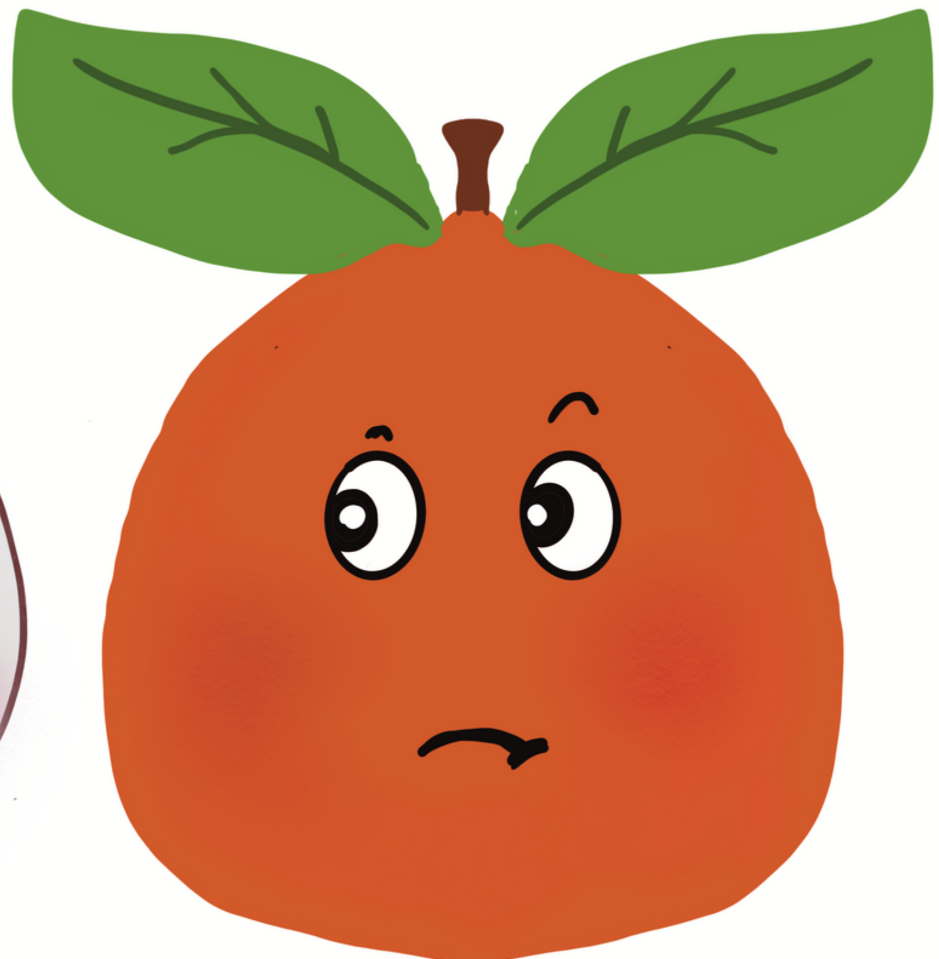

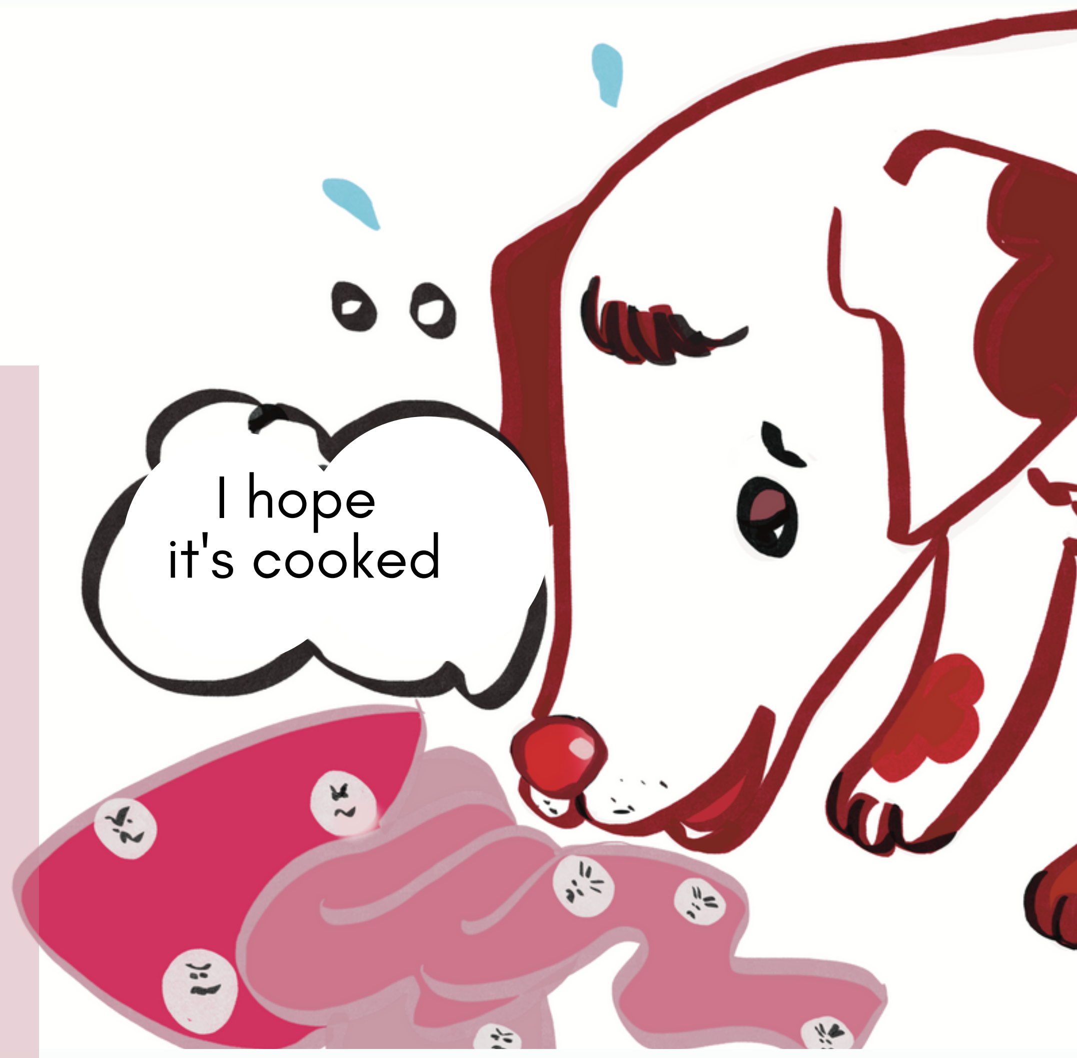

The dog is the major definitive host for transmission of cystic echinococcosis, and it gets infected by consumng the raw organs of animals infected with *E. granulosus* cysts.
The tapeworm requires 35 to 40 days to mature into an adult, with no symptoms in the host and allowing the parasitism to frequently go unnoticed. *E. granulosus* can survive between 6 and 20 months.
The dog's faeces contain 100 to 1,500 tiny eggs (30-50 × 22-44 m) that are released into the environment. Along with the faeces, parts of mature proglottids are also shed, which are loaded with infectious eggs that can contaminate food for humans, such as fruits and vegetables. In addition to direct input, grasses and waters can also be contaminated through passive disseminators such as animals, birds, insects, earthworms, and mollusks, as well as through wind, rain, and waterways.

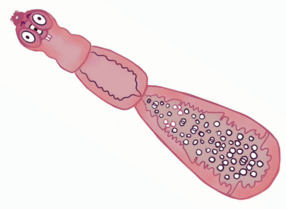

ID card of the parasite
Phylum: Plathelminths
Class: Cestoda
Order: Cyclophyllidei
Family: Taenidae
Genus: Echinococcus
Species: E. granulosus sensu latu
Size: 2-7 millimeters
Characteristics: the head, called the scolex, is studded with four suckers and a rostellum with a double crown of hooks with which it anchors itself firmly to the walls of the dog's intestine. The body consists of four segments, called proglottids

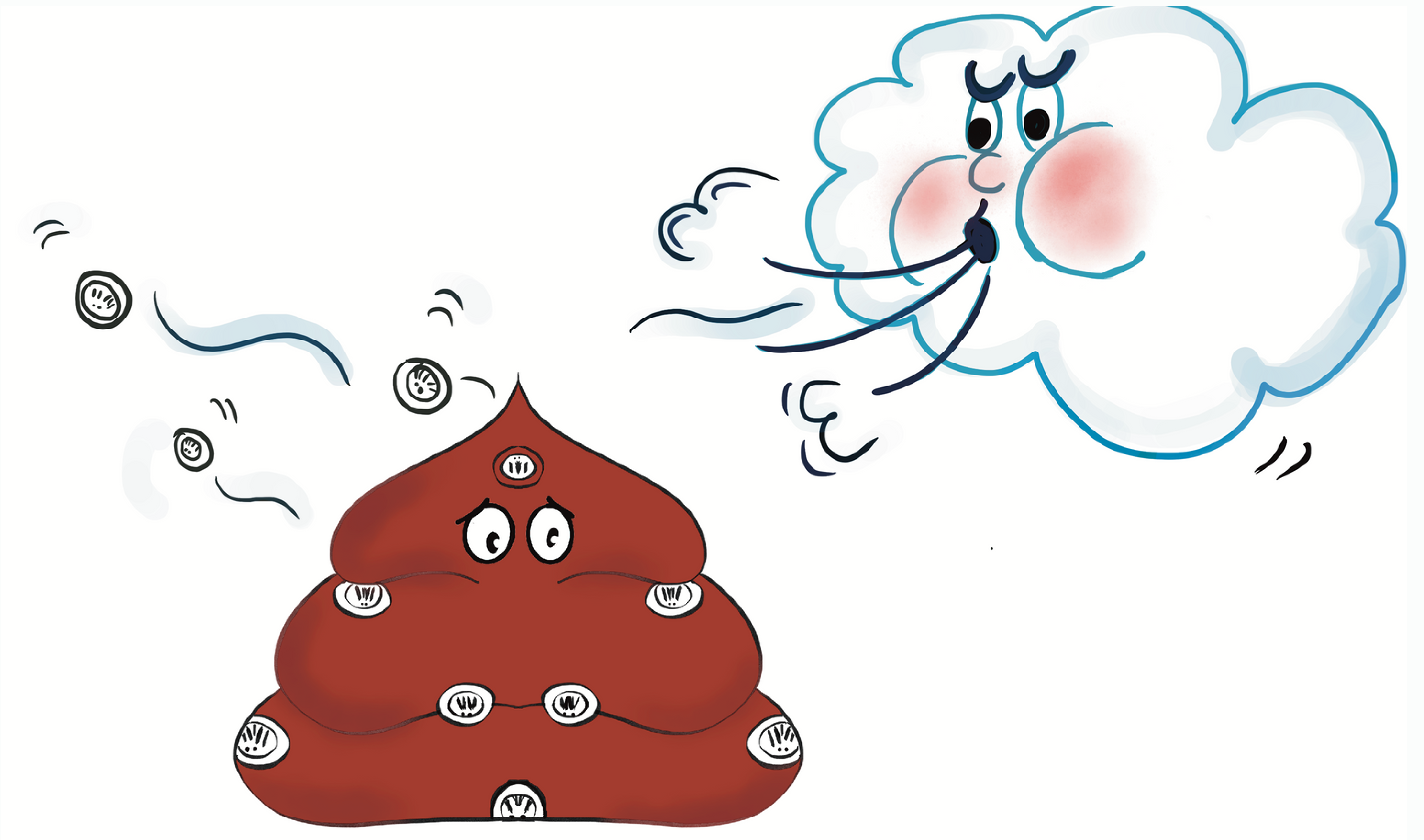

Not all dogs are infected by *Echinococcus*, but only those that eat raw meat or infected animal offal. It is advisable to take the dog for a periodic checkup with the veterinarian, who will conduct laboratory tests and can prescribe anthelmintics where necessary.

Shepherd dogs where extensive grazing is practiced and where family slaughter is not subject to regular state veterinary monitoring are at the highest risk of infection.

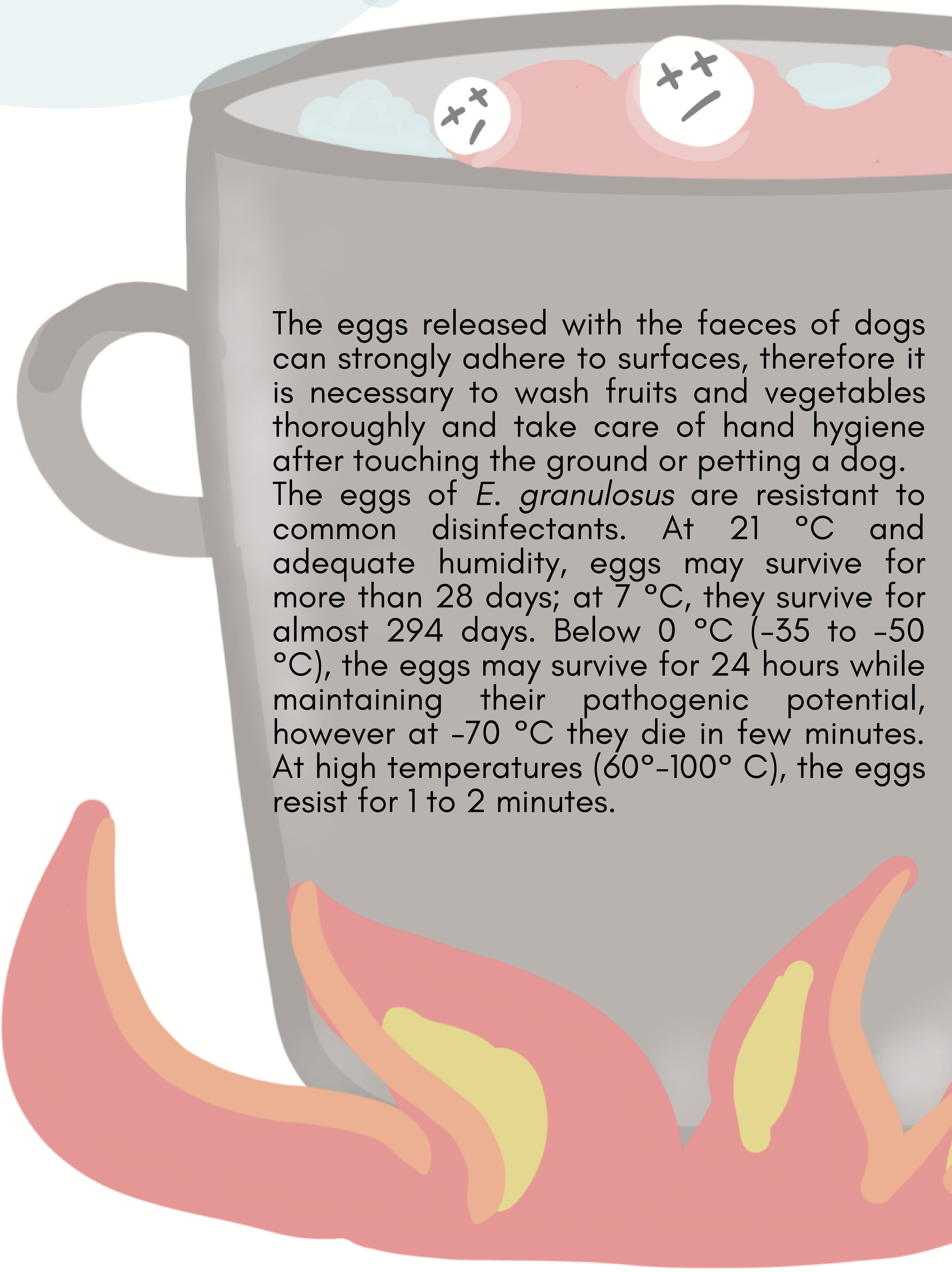

The eggs released with the faeces of dogs can strongly adhere to surfaces, therefore it is necessary to wash fruits and vegetables thoroughly and take care of hand hygiene after touching the ground or petting a dog.
The eggs of *E. granulosus* are resistant to common disinfectants. At 21 °C and adequate humidity, eggs may survive for more than 28 days; at 7 °C, they survive for almost 294 days. Below 0 °C (-35 to -50 °C), the eggs may survive for 24 hours while maintaining their pathogenic potential, however at -70 °C they die in few minutes. At high temperatures (60°-100° C), the eggs resist for 1 to 2 minutes.

How to prevent cystic echinococcosis

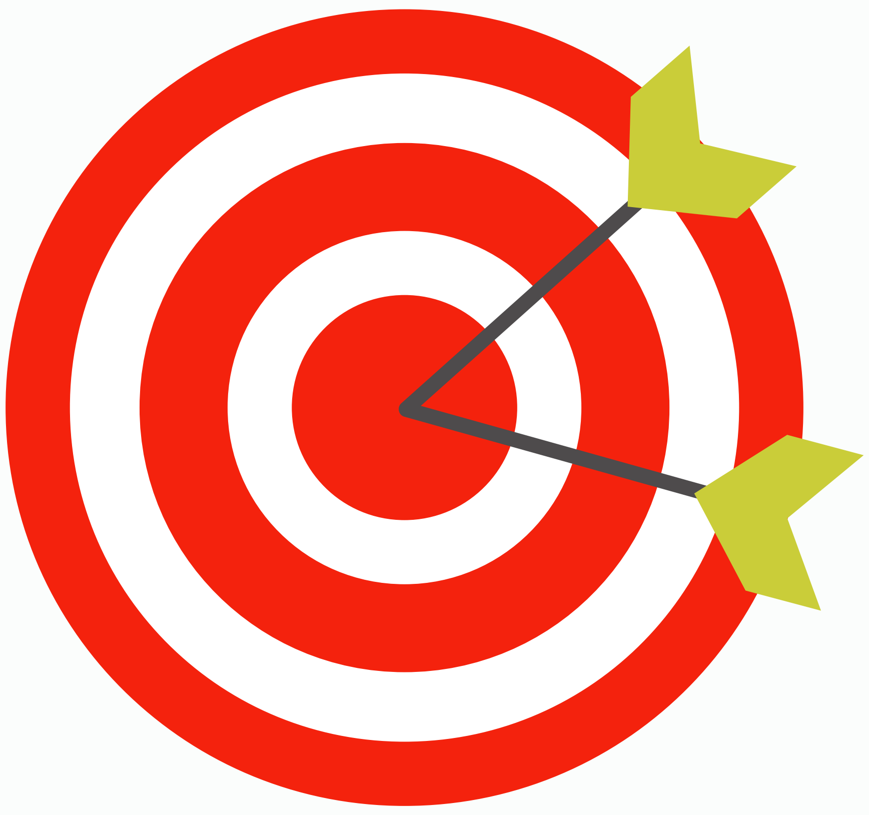

Despite considerable efforts to develop appropriate and effective methods of diagnosis, control, and prevention, this disease remains to date one of the major medical and veterinary health problems in many areas of the world.
Cystic echinococcosis is transmitted to animals and humans by the fecal-oral route, from dogs infected by the adult tapeworm; hence, primary prevention is one of the most important strategies.

Our advice:

- register your dog with the dog registry by having it microchipped at the Veterinary Service,
- collect and dispose of your dog's feces by following the municipality's waste collection rules,
- take your dog to veterinarian for routine coprological and general examination
- wash your hands thoroughly with soap, especially if you have touched the ground or petted an animal,
- wash fruits and vegetables thoroughly with running water,
- feed your dog well-cooked meat and viscera.

Graphic design Antonio Varcasia
illustrations by Francesca Porcu
english text version Fahad Ahmed

keep in touch:
facebook.com/parassitologia
instagram.com/vetparasitology
youtube.com/user/Parassitologia
https://www.parassitologia.com

vetoquinol
ACHIEVE MORE TOGETHER
