# Supplementary material for: 'Fight the parasite': raising awareness of cystic echinococcosis in primary school children in endemic countries
Source: Parasit Vectors. 2022 Dec 2;15:449. doi: 10.1186/s13071-022-05575-2 (PMC9717558; doi:10.1186/s13071-022-05575-2)
Supplement: Supplementary file 3 — Additional file 3: Movie file S1. “Fight the parasite” cartoon video, link and QR code, showing CE transmission and prevention in an entertaining narrative form. [file 13071_2022_5575_MOESM3_ESM.docx]

**Additional file 3**: Movie file S1: “Fight the parasite” cartoon video

<https://youtu.be/XTf3fTtmfA8>
