# Supplementary material for: 'Fight the parasite': raising awareness of cystic echinococcosis in primary school children in endemic countries
Source: Parasit Vectors. 2022 Dec 2;15:449. doi: 10.1186/s13071-022-05575-2 (PMC9717558; doi:10.1186/s13071-022-05575-2)
Supplement: Supplementary file 4 — Additional file 4: Text S3. Questionnaire to assess students’ knowledge of CE and quiz solutions with the right answers, edited in English and Spanish. [file 13071_2022_5575_MOESM4_ESM.pdf]

Quiz 1

Please answer these questions BEFORE
you fill in the educational booklet

Circle if you think the statement is true (T), false (F) or don't know (DK)

Question 1: T F DK
Dogs become infected by *Echinococcus* by eating cooked sheep offals

Question 2: T F DK
You could get infected by touching your dog's feces

Question 3: T F DK
Washing fruits and vegetables well keeps you protected from the parasite

Question 4: T F DK
There is no medicine for dogs against *Echinococcus*

Question 5: T F DK
Washing hands after touching a dog can help you not get the parasite.

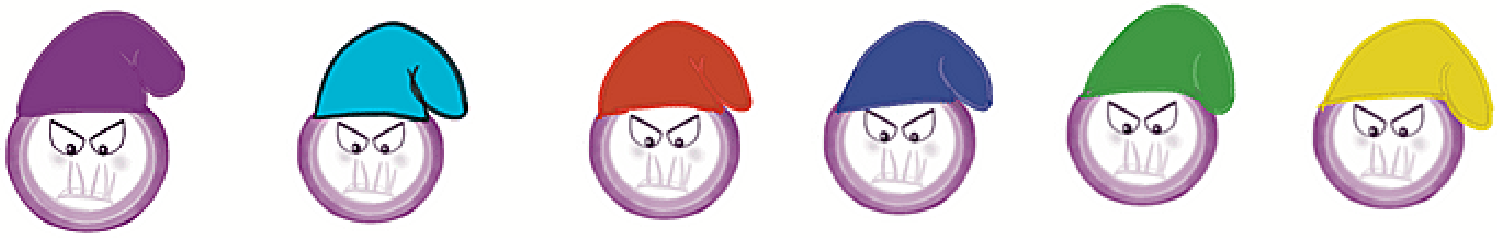

Fight the parasite!

An edutainment project for Cystic Echinococcosis
awareness in primary schools of endemic countries

Quiz 2

Please answer these questions AFTER
you fill in the educational booklet

Circle if you think the statement is true (T), false (F) or don't know (DK)

Question 1: T F DK
Dogs become infected by *Echinococcus* by eating cooked sheep offals

Question 2: T F DK
You could get infected by touching your dog's feces

Question 3: T F DK
Washing fruits and vegetables well keeps you protected from the parasite

Question 4: T F DK
There is no medicine for dogs against *Echinococcus*

Question 5: T F DK
Washing hands after touching a dog can help you not get the parasite.

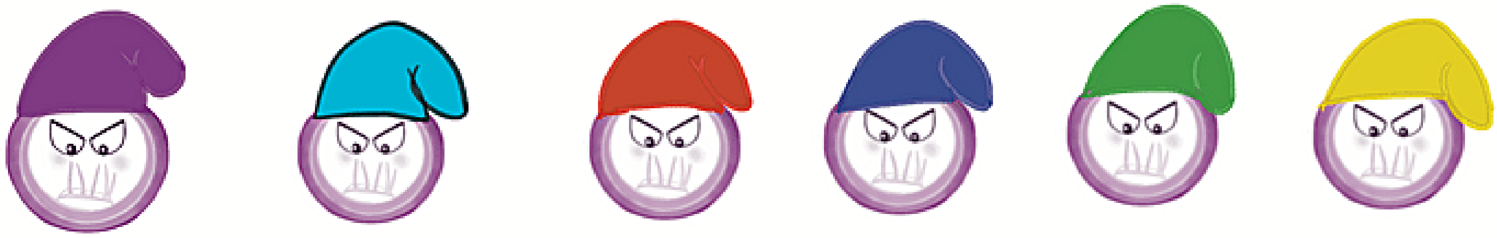

Fight the parasite!

An edutainment project for Cystic Echinococcosis
awareness in primary schools of endemic countries

Quiz

solutions

Circle if you think the statement is true **T**, false **F** or don't know **DK**

Question 1: T **F** DK
Dogs become infected by *Echinococcus* by eating cooked sheep offals

Question 2: **T** F DK
You could get infected by touching your dog's feces

Question 3: **T** F DK
Washing fruits and vegetables well keeps you protected from the parasite

Question 4: T **F** DK
There is no medicine for dogs against *Echinococcus*

Question 5: **T** F DK
Washing hands after touching a dog can help you not get the parasite.

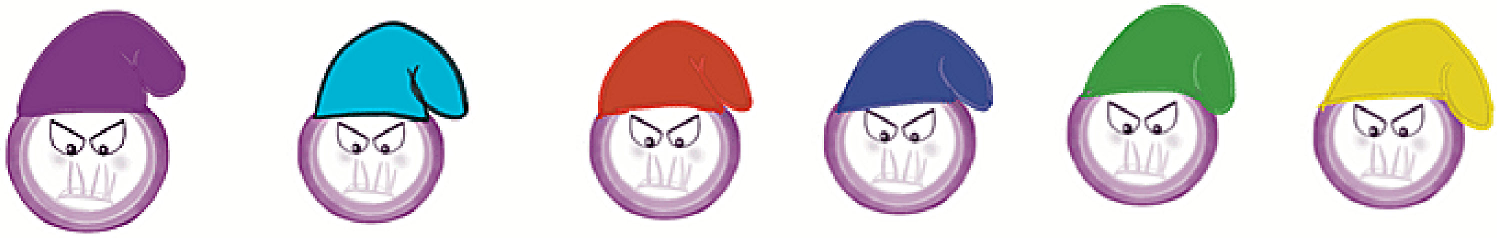

Fight the parasite!

An edutainment project for Cystic Echinococcosis
awareness in primary schools of endemic countries
