# Supplementary material for: 'Fight the parasite': raising awareness of cystic echinococcosis in primary school children in endemic countries
Source: Parasit Vectors. 2022 Dec 2;15:449. doi: 10.1186/s13071-022-05575-2 (PMC9717558; doi:10.1186/s13071-022-05575-2)

**Additional file 5:** Movie files S2. Link to slideshow of artwork made by children

[**https://youtu.be/9kWQ7JzIlLM**](https://youtu.be/9kWQ7JzIlLM)


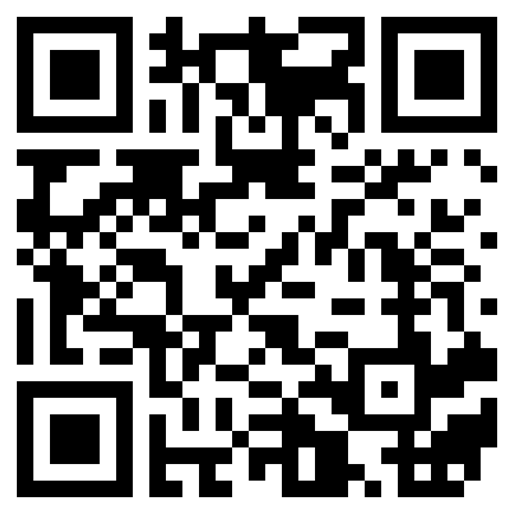

Supplement: Supplementary file 5 — Additional file 5: Movie file S2. Link to slideshow of artwork made by children. [file 13071_2022_5575_MOESM5_ESM.docx]
