# Supplementary material for: 'Fight the parasite': raising awareness of cystic echinococcosis in primary school children in endemic countries
Source: Parasit Vectors. 2022 Dec 2;15:449. doi: 10.1186/s13071-022-05575-2 (PMC9717558; doi:10.1186/s13071-022-05575-2)
Supplement: Supplementary file 6 — Additional file 6: Text S4. Instructions for teachers to carry out the project in the classroom. [file 13071_2022_5575_MOESM6_ESM.docx]

**Additional file 6: Text S4. Instructions for teachers to carry out the project in the classroom**

Teachers who would like to carry out the project in their classroom, could follow the instructions below. In attachment you will find all the material needed for making the project. All the contents have been created by the authors of the present paper and can be used without any limitation in all countries of the world. All the material was edited in English and Spanish language, for a wider and easier use in the Spanish-speaking Countries. The only mandatory thing is to provide a citation of the paper during presentation and in any work carried out (Porcu et al. 2022, Fight the parasite, an edutainment project for Cystic Echinococcosis awareness in primary schools of endemic countries; XXXYY).

At the beginning of the meeting the quiz 1 (Additional file 4: Text S3) will be handed out to students. Download and hand out the comic educational booklet (Additional file 1: Text S1) or show it using the whiteboard and encourage discussion about their ideas. Later, a kind of small lecture on the epidemiology and biological cycle of *E. granulosus* will be administered to the students, using the information contained in the Teacher's guidebook (Additional file 2: Text S2). Give space for children creativity by letting them realize drawings about cystic echinococcosis. Show the cartoon video (Additional file 3: Movie files S1). At the end of the educational meeting deliver quiz 2 (Additional file 4: Text S3) for follow-up and check the right answers (Additional file 4: Text S3).
